# Deletion of *sf3b4* causes splicing defects and gene dysregulation that disrupt craniofacial development and survival

**DOI:** 10.1242/dmm.052169

**Published:** 2025-03-24

**Authors:** Casey Griffin, Kelsey Coppenrath, Doha Khan, Ziyan Lin, Marko Horb, Jean-Pierre Saint-Jeannet

**Affiliations:** ^1^ Department of Molecular Pathobiology, College of Dentistry, New York University, New York, NY 10010, USA; ^2^ Eugene Bell Center for Regenerative Biology and Tissue Engineering and National Xenopus Resource, Marine Biological Laboratory, Woods Hole, MA 02543, USA; ^3^ Applied Bioinformatics Laboratory, NYU Grossman School of Medicine, New York, NY 10010, USA

**Keywords:** SF3B4, Nager syndrome, *Xenopus tropicalis*, Neural crest, Splicing

## Abstract

Nager and Rodriguez syndromes are rare craniofacial and limb disorders characterized by midface retrusion, micrognathia, absent thumbs and radial hypoplasia. These disorders result from haploinsufficiency of SF3B4 (splicing factor 3b, subunit 4), a component of the pre-mRNA spliceosomal machinery. Although the spliceosome is present and functions in all cells of the body, most spliceosomopathies – including Nager and Rodriguez syndromes – are cell- or tissue-specific in their pathology. To understand the pathomechanism underlying these conditions, we generated a *Xenopus tropicalis sf3b4* mutant line using CRISPR/Cas9 gene-editing technology. Homozygous deletion of *sf3b4* is detrimental to the development of cranial neural crest (NC)-derived cartilage progenitors. Temporal RNA-sequencing analyses of mutant embryos identified an increase in exon-skipping events, followed by important transcriptional changes associated with an enrichment for terms consistent with defects in NC cell migration and survival. We propose that disruption of these processes may underly the pathogenesis of Nager and Rodriguez syndromes.

## Teaser

**Editor's choice:** Characterization of a *Xenopus tropicalis sf3b4* mutant line generated using CRISPR/Cas9 gene-editing technology as a novel model to understand the pathogenesis of Nager and Rodriguez syndromes.

## INTRODUCTION

Nager syndrome (OMIM #154400) is a form of acrofacial dysostosis (AFD), a rare type of disorder characterized by malformations of the craniofacial skeleton and limbs (Halal et al., 1983). Individuals with Nager syndrome specifically present with downslanting palpebral fissures, midface retrusion, micrognathia, defective middle ear ossicles, and hypoplastic or absent thumbs (Trainor and Andrews, 2013). Nager syndrome is a rare disorder, with approximately 100 reported cases ever, worldwide. The craniofacial skeletal structures affected in Nager syndrome are neural crest (NC)-derived.

The NC is an embryonic cell type unique to vertebrates. These cells arise from the neural plate border region of developing embryos, undergo an epithelial-to-mesenchymal transition, migrate throughout the embryo, and eventually give rise to numerous cell types and structures, including melanocytes, the peripheral nervous system in the trunk and most of the craniofacial skeleton in the head. A subdomain of the NC, known as the cranial NC, originating from the mesencephalon and rhombencephalon, migrates as streams to populate the branchial arches. The branchial arches are composed of NC cells surrounding a core of mesoderm and give rise to the majority of the differentiated tissues of the head and neck. The mesoderm-derived cells form the musculature of the head and the endothelial cells of the arch arteries, whereas NC cells develop into craniofacial skeletal elements specific for each branchial arch (Trainor and Krumlauf, 2000). The craniofacial structures affected in Nager syndrome are primarily derived from the first and second branchial arches (Passos-Bueno et al., 2009).

The major cause of Nager syndrome, in approximately 60% of cases, is haploinsufficiency of the splicing factor 3b, subunit 4 (*SF3B4*) gene (Bernier et al., 2012; Czeschik et al., 2013; Petit et al., 2014). The remaining 40% of cases are of unknown cause. *SF3B4* pathogenic variants are predicted to encode truncated proteins with or without altered amino acid sequences at the C-terminus end (Bernier et al., 2012), suggesting that Nager syndrome results from haploinsufficiency. Most cases are sporadic, and both autosomal-dominant and autosomal-recessive inheritances have been reported (Chemke et al., 1988; Hall, 1989). Rodriguez syndrome (OMIM #201170) is another condition due to mutations in *SF3B4* (Drivas et al., 2019). Although this syndrome is characterized by similar craniofacial abnormalities as Nager syndrome, the defects are usually more severe and involve lower limb anomalies, cardiac defects and arhinencephaly (Rodríguez et al., 1990). *SF3B4* encodes SAP49, a component of the U2 subunit of the major spliceosome (Will and Luhrmann, 2011). The spliceosome is a complex made up of RNAs and proteins that functions to identify non-coding introns in precursor messenger-RNA (pre-mRNA) and promote accurate splicing at the surrounding splice sites. Recognition of the 5′ and 3′ splice sites, as well as proper binding to the pre-mRNA and other parts of the spliceosome complex are required for the splicing process to occur. At each step of the splicing process, different sets of small nuclear ribonucleoproteins (snRNPs) are recruited, and the combination of RNA, small nuclear RNA (snRNA), snRNP and non-snRNP protein interactions allow for splicing to occur at the proper location (Will and Luhrmann, 2011). SF3B4 contains two RNA recognition motifs at its N-terminal end through which it binds upstream to the branch site in the intronic region of the pre-mRNA, where it helps tether the U2 complex to the branch site.

Previous work in mouse models has shown that a loss of SF3B4 affects the axial skeleton and the forebrain, with no apparent craniofacial defects (Yamada et al., 2020; Kumar et al., 2023). This phenotype was associated with a disruption in Hox gene expression and aberrant splicing of several chromatin remodelers (Kumar et al., 2023). In *Xenopus laevis*, morpholino-mediated knockdown of *sf3b4* results in a loss of NC gene expression and causes a reduction or loss of craniofacial cartilages at tadpole stages through a mechanism that involves increased apoptosis (Devotta et al., 2016). A mutation in *sf3b4* in zebrafish has been reported to result in a phenotype reminiscent of retinitis pigmentosa, a spliceosomopathy affecting the photoreceptors in the retina (Ulhaq et al., 2023). Despite these efforts, for the most part the precise mechanisms underlying the pathogenesis of Nager syndrome remain largely unknown. It is especially puzzling that variants of a core component of this largely ubiquitous cellular machinery result in such an exquisitely cell type- and lineage-specific defect.

Here, we report the characterization of a *Xenopus tropicalis sf3b4* mutant line generated using CRISPR/Cas9 gene-editing technology. We show that in *sf3b4* mutants NC induction is unaffected. However, at the tailbud stage these animals show NC migration defects and increased apoptosis in the head region. Although homozygous animals fail to survive beyond the tadpole stage, heterozygous animals are phenotypically largely indistinguishable from wild-type tadpoles. RNA-sequencing (RNA-seq) and Gene Ontology (GO) analyses of embryos at different developmental stages reveal an increase in aberrant splicing events, followed by a massive dysregulation of gene expression in homozygous animals, with an enrichment for terms consistent with defects in NC cell migration and survival as the possible underlying causes of Nager syndrome.

## RESULTS

### Developmental expression of *sf3b4*

**Fig. 1.**
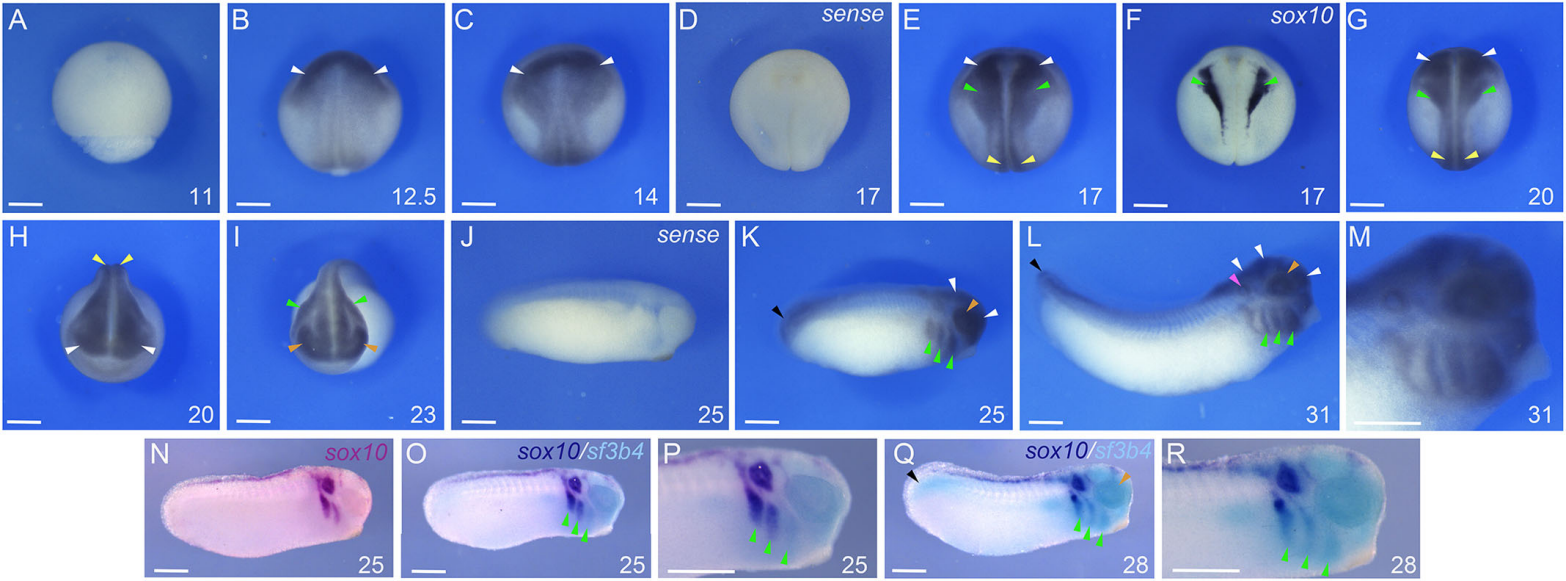
**Developmental expression of *Xenopus tropicalis sf3b4.*** (A-M) *sf3b4* is not expressed at the mid-gastrula stage (A) but is first detected at the end of gastrulation in the dorsal ectoderm (B). During neurulation (C,E-H), *sf3b4* transcripts are detected in the developing neural plate/tube and neural crest (NC)-forming regions, where it overlaps with *sox10* (F). At tailbud stages (K-M), *sf3b4* expression persists in the brain, eyes, migrating NC cells, and is also detected in the otic vesicles and the tailbud. Anterior neural plate/developing brain (white arrowheads), prospective spinal cord (yellow arrowheads), neural crest (green arrowheads), developing eyes (brown arrowheads), otic vesicle (magenta arrowhead) and tailbud (black arrowheads) are indicated. Embryos hybridized with a sense control are shown for stage 17 (D) and stage 25 (J). (N-R) Two-color WMISH for *sox10* and *sf3b4* at stage 25 (O,P) and stage 28 (Q,R), reveals co-expression of these genes in the migrating NC cells populating the posterior branchial arches. At these stages, *sox10* expression is progressively downregulated in the most anterior NC streams (N). (A-G) Dorsal views, anterior to top. (H,I) Frontal views, dorsal to top. (J-R) Lateral views, anterior to right, dorsal to top. The embryonic stages (NF) are indicated in the lower right corner of each panel. Images are representative of 25 samples. Scale bars: 150 µm.

We first analyzed the developmental expression of *sf3b4* in *Xenopus tropicalis* embryos using whole-mount *in situ* hybridization (WMISH) with a short chromogenic reaction to highlight areas of *sf3b4* enrichment. *sf3b4* transcripts were first detected at the end of gastrulation [Nieuwkoop and Faber (NF) stage 12.5] in the dorsal anterior ectoderm (Fig. 1A,B). At the early neurula stage (NF stage 14), the *sf3b4* expression domain encompassed the neural plate and the neural plate border (Fig. 1C). As neurulation proceeded (NF stage 17), *sf3b4* transcripts remained broadly enriched dorsally, including the prospective brain and spinal cord (Fig. 1E), and in a domain that overlapped with the *sox10* expression domain in the NC territory (Fig. 1F). As development continued, *sf3b4* was detected in the head region, including the developing brain, spinal cord, eyes and migrating NC cells (Fig. 1G-I). At tailbud stages (NF stages 25-31), *sf3b4* expression was largely confined to the brain, eyes, otic vesicles, branchial arches and the tailbud (Fig. 1K-M). Control sense probe at stage 14 (Fig. 1D) and stage 25 (Fig. 1J) did not show any signal. Two-color WMISH for *sox10* and *sf3b4*, revealed substantial overlap between these two genes in the migrating NC cells populating the posterior branchial arches (Fig. 1N-R). The expression pattern of *X. tropicalis sf3b4* was similar to that of *X. laevis sf3b4*, with an enrichment dorsally and anteriorly at neurula stage, and in the branchial arches at the tailbud stage (Devotta et al., 2016). Although the expression pattern of *SF3B4* in human embryos remains uncharacterized, in the mouse *Sf3b4* expression is enriched in the cranial region, including the brain and the branchial arches, as well as the limb bud mesenchyme and the heart at embryonic day 10.5 (Yamada et al., 2020). A longer chromogenic reaction revealed expression of *sf3b4* throughout the embryo, including those regions reported in the mouse (C.G., unpublished observations). The *sf3b4* expression domain over time encompasses regions of the frog embryo that are broader than the NC territory, therefore this expression pattern cannot account for the cell type-specific effect of *sf3b4* mutation in cranial NC and its derivatives.

### Generation of a *Xenopus tropicalis* CRISPR/Cas9 *sf3b4* mutant line

**Fig. 2.**
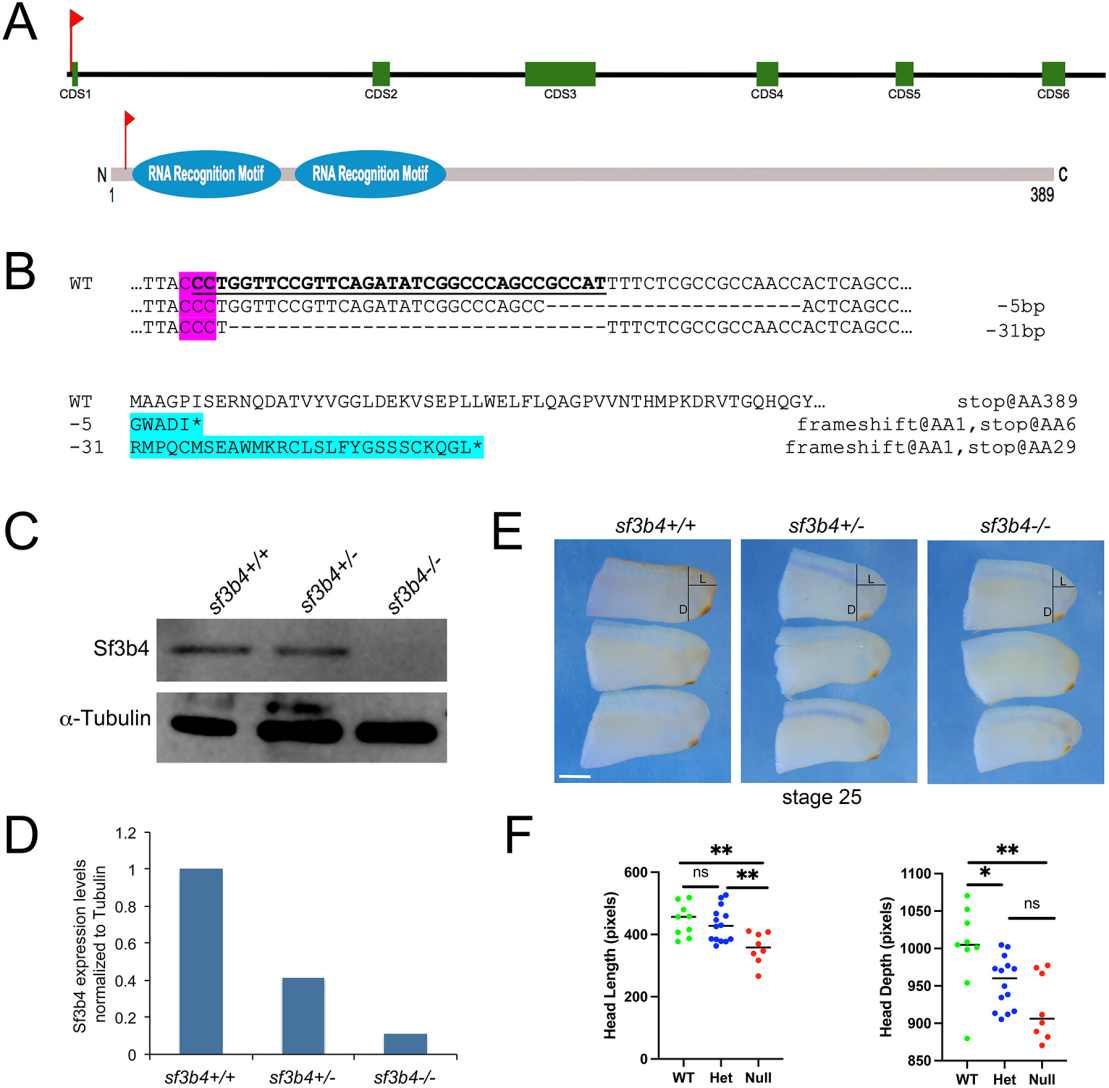
**Generation of a *Xenopus tropicalis* CRISP/Cas9 *sf3b4* mutant line.** (A) Schematic of the genomic *sf3b4* locus, and Sf3b4 protein. The relative position of the sgRNA target sequence is indicated (red flags). CDS1-CDS6 represent exons 1-6. (B) Alignment of wild-type (WT) and −5 bp or −31 bp mutated nucleotide sequences. The PAM sequence is indicated (magenta). A frameshift in the mutant nucleotide sequence results in an incorrect protein sequence with an early stop (asterisk) after amino acids 5 and 28, respectively (cyan). Shown underlined is the targeted nucleotide sequence. (C) Western blot analysis of protein extracts from WT (+/+), heterozygote (+/−) and homozygote (−/−) F2 tailbud-stage embryos (NF stage 25), using an anti-Sf3b4 antibody. α-Tubulin is shown as a loading control. *n*=10. (D) ImageJ quantification of the western blot. (E) *sf3b4* mutant embryos at stage 25 have reduced head length and width compared to WT. The distances measured to quantify head length (L) and depth (D) are indicated on the embryo at the top for each genotype. Lateral views, anterior to right, dorsal to top. (F) Graph plotting the length and width for all three genotypes. ns, not significant. *P<0.05, **P<0.01 (Welch's two-tailed unpaired *t*-test). *n*=9 (WT), 14 (Het), 8 (Null). Scale bars: 150 µm.

To generate this custom mutant line, five sgRNAs were synthesized targeting the first three exons of *X. tropicalis sf3b4* (Fig. 2A). A mutation in this region has been described in individuals with Nager syndrome from three unrelated families; it is predicted to abolish the methionine initiation codon resulting in a complete lack of protein product (Bernier et al., 2012). Of the 50 embryos injected at the one-cell stage, 20 reached adulthood. After reaching sexual maturity, one female was outcrossed to a wild-type (WT) male and tested for germline transmission. Genotyping of the corresponding F1 embryos showed both −5 bp and −31 bp mutations. The remaining F1 embryos were reared and genotyped as adults by hindlimb web punch sampling. Out of 50 F1 adults, 15 had a confirmed −31 bp mutation. The −31 bp heterozygous F1 *sf3b4* adults were intercrossed to generate F2 mutants used for this study. The −31 bp mutation disrupts the start codon of Sf3b4, resulting in a frameshift at the first amino acid, and introducing an early stop codon 29 amino acids downstream (Fig. 2B).

Western blot analysis of stage 25 WT, heterozygous (Het), and homozygous (Null) mutant embryos showed that Sf3b4 protein expression levels are consistent with each genotype and the number of intact copies of the gene (Fig. 2C,D; Fig. S1). By gross morphology, mutant embryos at the tailbud stage (NF stage 25) displayed reduced head length and depth, a phenotype that was more pronounced in Null mutants (Fig. 2E,F).

### Characterization of the *Xenopus tropicalis* CRISPR/Cas9 *sf3b4* mutant embryos

**Fig. 3.**
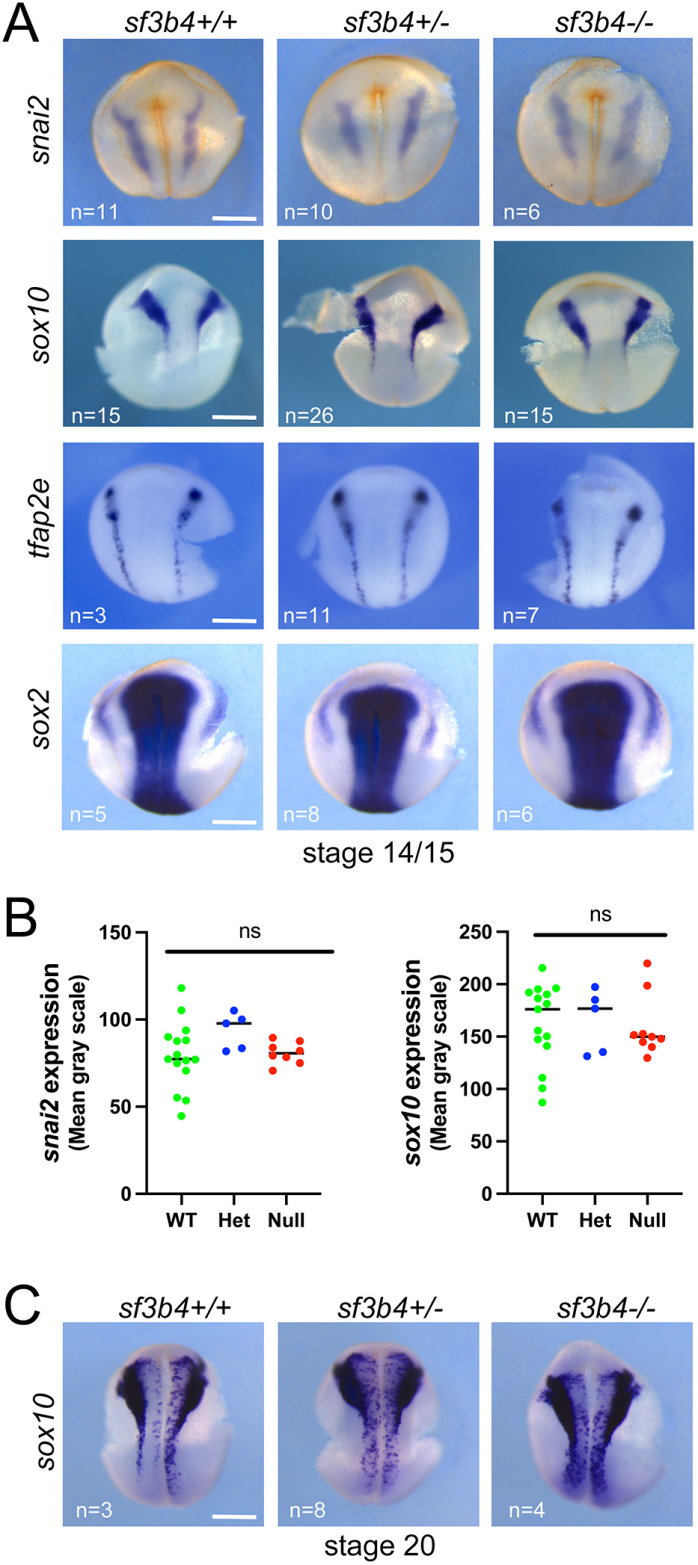
**Molecular characterization of *sf3b4* mutant embryos at neurula stages.** (A) At NF stage 14/15, the expression of *snai2*, *sox10* and *tfap2e* in NC progenitors is largely unaltered in all three genotypes: *sf3b4* WT (+/+), heterozygote (+/−) and homozygote (−/−). The neural plate expression of *sox2* is also unaffected. (B) ImageJ quantification of *snai2* and *sox10* WMISH signal. ns, not significant. Welch's two-tailed unpaired *t*-test (see labeling within panel images for *n* numbers). (C) At the end of neurulation, NF stage 20, the expression of *sox10* is largely unaltered in all three genotypes. (A,C) Dorsal views, anterior to top. Scale bars: 300 µm.

**Fig. 4.**
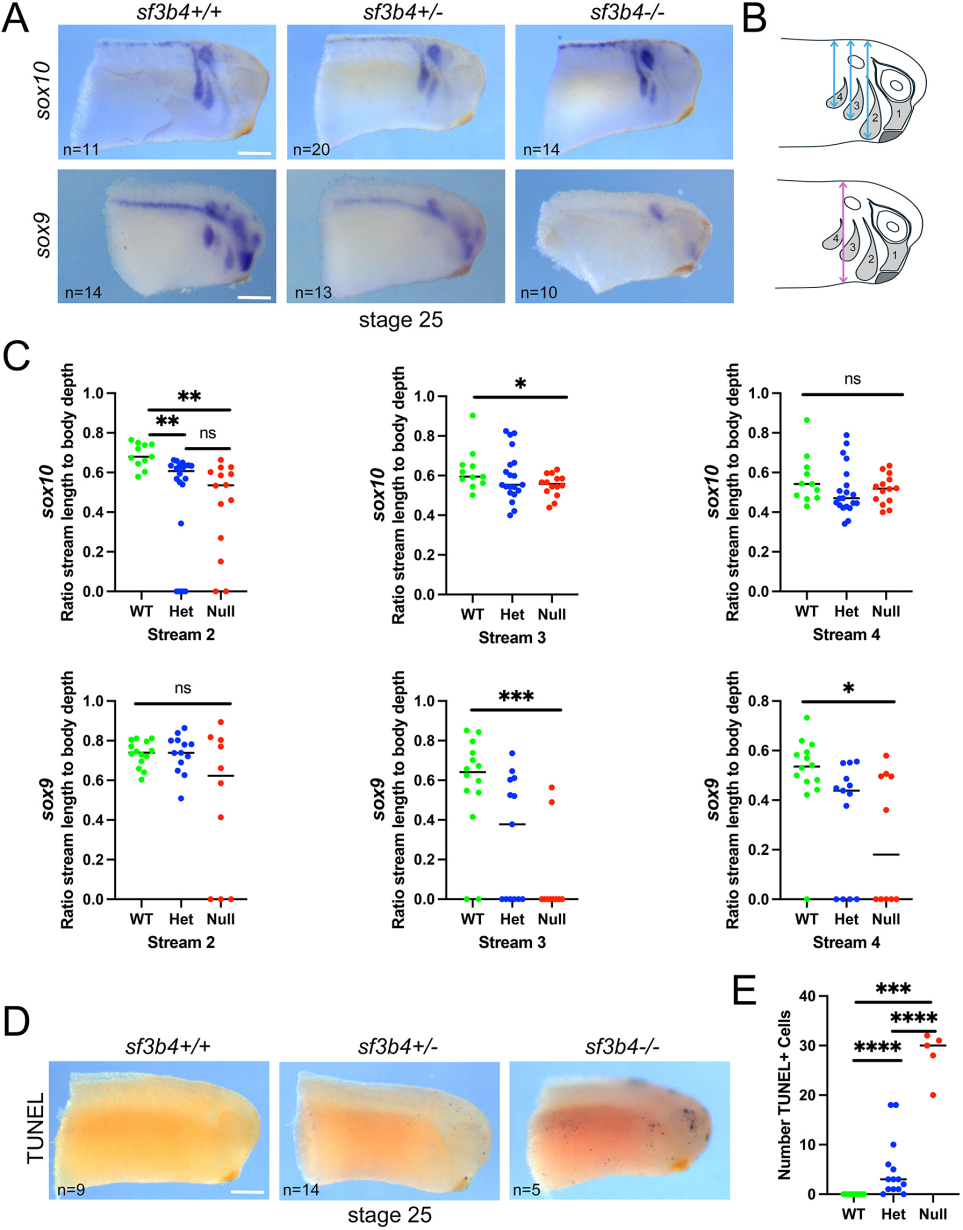
**Molecular characterization of *sf3b4* mutant embryos at tailbud stage.** (A) At NF stage 25, the expression of *sox9* and *sox10* in the NC streams is reduced or absent in heterozygous and null embryos. *sf3b4* WT (+/+), heterozygote (+/−) and homozygote (−/−). (B) Diagrams illustrating the position of the NC streams (1-4), and the distance measured to quantify NC streams length (top, blue lines) and its normalization to body depth (bottom, magenta line). (C) Quantification of stream length compared to full body width of embryos. ns, not significant. *P<0.05, **P<0.01, ***P<0.001 (Welch's two-tailed unpaired *t*-test; see labeling within panel images for *n* numbers). (D) TUNEL staining of NF stage 25 embryos. (E) Quantification of TUNEL staining. ***P<0.001, ****P<0.0001 (Welch's two-tailed unpaired *t*-test; see labeling within panel images for *n* numbers). (A,D) Lateral views, anterior to right, dorsal to top. Scale bars: 150 µm.

Because NC-derived craniofacial skeletal elements are primarily affected in Nager syndrome, we analyzed by WMISH the expression of genes to visualize the NC at pre-migratory (*snai2*, *sox10*, *tfap2e*) and migratory (*sox9*, *sox10*) stages. Across all three genotypes, we found no difference in the expression of *snai2*, *sox10* and *tfap2e* at early neurula stage (NF stage 14/15; Fig. 3A,B; Table S1). Because *sf3b4* was also expressed in the neural plate (Fig. 1), we examined the expression of *sox2* at early neurula stage. This gene was unaffected in mutant (Het and Null) embryos compared to WT sibling controls (Fig. 3A; Table S1). Upon closure of the neural plate (NF stage 20), *sox10* expression pattern in the NC territory remained unchanged across genotypes (Fig. 3C; Table S1). At the migratory stage (NF stage 25) there was a notable phenotype in Het and Null mutant embryos characterized by a decrease in the length of a subset of cranial NC streams, as evidenced by *sox9* and *sox10* expression (Fig. 4A,B; Table S1). Quantification of the phenotype showed a statistically significant reduction of the NC stream length (streams 2 and 3 for *sox10*, and streams 3 and 4 for *sox9*) in Het and Null embryos compared to WT siblings (Fig. 4C). The differential effect on *sox9* and *sox10* may reflect gene-specific sensitivity to the loss of *sf3b4*. To understand the mechanism driving this phenotype, we performed terminal deoxynucleotidyl transferase dUTP nick end labeling (TUNEL) staining at different developmental stages. We found no change in apoptosis in *sf3b4* mutants at NF stage 20 (Fig. S2), which correlates with robust expression of *sox10* in the pre-migratory NC territory at this stage (Fig. 3C). However, at the migratory stage (NF stage 25), there was a significant increase in apoptosis in the head region of Het and Null embryos, with a greater number of TUNEL-positive cells in the Null compared to the Het animals (Fig. 4D,E). These results suggest that although NC cell formation is not affected at the neural plate border in *sf3b4* mutants, their migration in the branchial arches is impaired.

### Characterization of *Xenopus tropicalis* CRISPR/Cas9 *sf3b4* mutant tadpoles

**Fig. 5.**
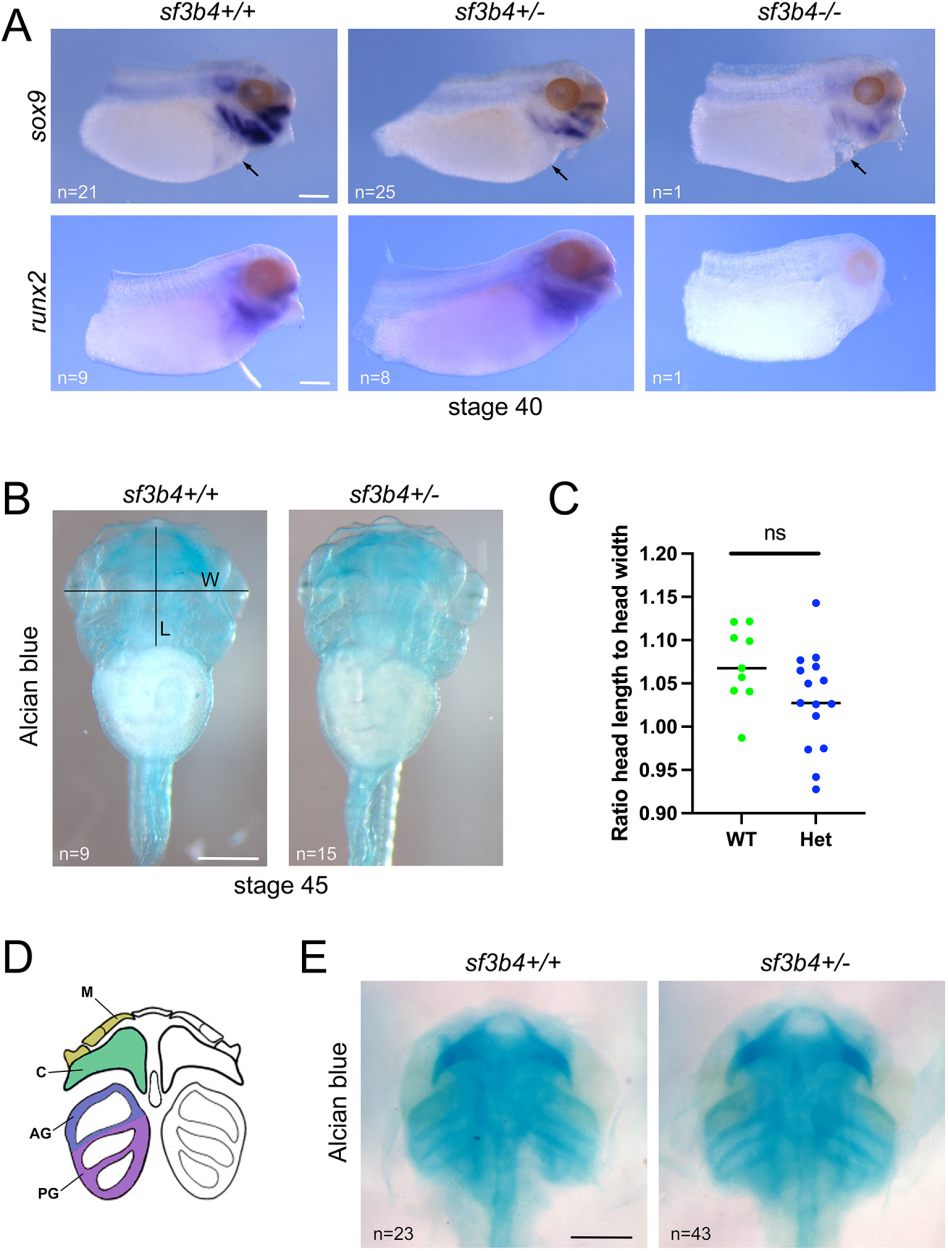
**Molecular characterization of the *sf3b4* mutant tadpoles.** (A) At NF stage 40, *sox9* and *runx2* expression in the branchial arches is severely reduced in Null embryos. Lateral views, anterior to right, dorsal to top. *sf3b4* WT (+/+), heterozygote (+/−) and homozygote (−/−). Scale bars: 150 µm. (B) Alcian Blue-stained tadpoles, NF stage 45. Ventral views, anterior to top. *sf3b4* WT (+/+) and heterozygote (+/−). Head width (W; distance from eyeball to eyeball) and head length (L; distance from the mouth to the gut) measurements are indicated. Scale bar: 500 µm. (C) Graph plotting the ratio head length to head width for WT and Het tadpoles. ns, not significant. Welch's two-tailed unpaired *t*-test. *n*=9 (WT), 15 (Het). (D) Diagram illustrating the NC-derived cranial cartilages at the tailbud stage (modified from Sadaghiani and Thiebaud, 1987). From anterior to posterior, Meckel's cartilage (M), ceratohyal cartilage (C), and anterior (AG) and posterior (PG) gill cartilages. (E) Ventral views of dissected craniofacial cartilages of *sf3b4* WT (+/+) and heterozygote (+/−) tadpoles. n, number of embryos analyzed per genotype. Scale bar: 500 µm.

We next analyzed mutant tadpoles at post-migratory stage (NF stage 40), when NC cells coalesce in the branchial arches into foci of cartilage precursors expressing *sox9* and *runx2*. We noticed that the number of *sf3b4* Null animals recovered at this stage was lower than the expected Mendelian ratio (Fig. S3), suggesting that the phenotype described at the migratory stage may not be fully compatible with survival. Our analysis of the *sf3b4* mutant tadpoles indicates that *sox9* expression is severely decreased in Null compared to Het and WT embryos (Fig. 5A; Table S1). *sox9* expression was, however, not completely lost in the mutants, suggesting that the subset of NC cells that migrates into the branchial arches can initiate a cartilage differentiation program. We also analyzed *runx2* expression at this stage, a marker for cartilage progenitors. We found that *runx2* expression was completely lost in the Null compared to Het and WT embryos (Fig. 5A; Table S1), again indicating that the formation of NC-derived cartilages is severely impaired in Null mutants. We also noticed by gross morphology that the few Null tadpoles that survived up to that stage appeared to present heart defects that were not observed in WT and Het animals (Fig. 5A). To investigate further a possible heart phenotype, we analyzed the expression of several genes that are crucial for cardiac development, namely *tbx5*, *tbx20* and *nkx2-5*, at multiple stages of development (stages 25, 30 and 35). Whereas *tbx5* and *nkx2-5* expression was largely unperturbed in Het and Null animals at stage 25 and stage 30, respectively, we observed a marked decreased expression of *nkx2-5* and *tbx20* in the Null embryos at stage 35 compared to Het and WT embryos (Fig. S4A). Furthermore, transcriptomic analysis of Het versus Null at stage 35 (see below) revealed an enrichment for terms such as ‘cardiac septum morphogenesis’ and ‘heart morphogenesis’ among the downregulated genes (Fig. S4B; Table S2), consistent with a role of Sf3b4 in cardiac development.

Finally, we analyzed the long-term consequences of *sf3b4* deletion on craniofacial cartilage formation at NF stage 45 by performing Alcian Blue staining (Fig. 5E). Interestingly, we did not recover any Null tadpoles at this stage; all genotyped animals were either WT or Het (Fig. S3), suggesting that the defects observed at earlier developmental stages are too severe for survival. Head cartilage staining of WT and Het tadpoles revealed that these structures are largely unaffected in Het tadpoles, with a slight decrease in the overall size of the head, which was not statistically significant (Fig. 5B,C). Dissected craniofacial cartilages of *sf3b4* Het tadpoles were largely indistinguishable from that of their WT siblings (Fig. 5D).

### Changes in gene expression associated with Sf3b4 loss of function

**Fig. 6.**
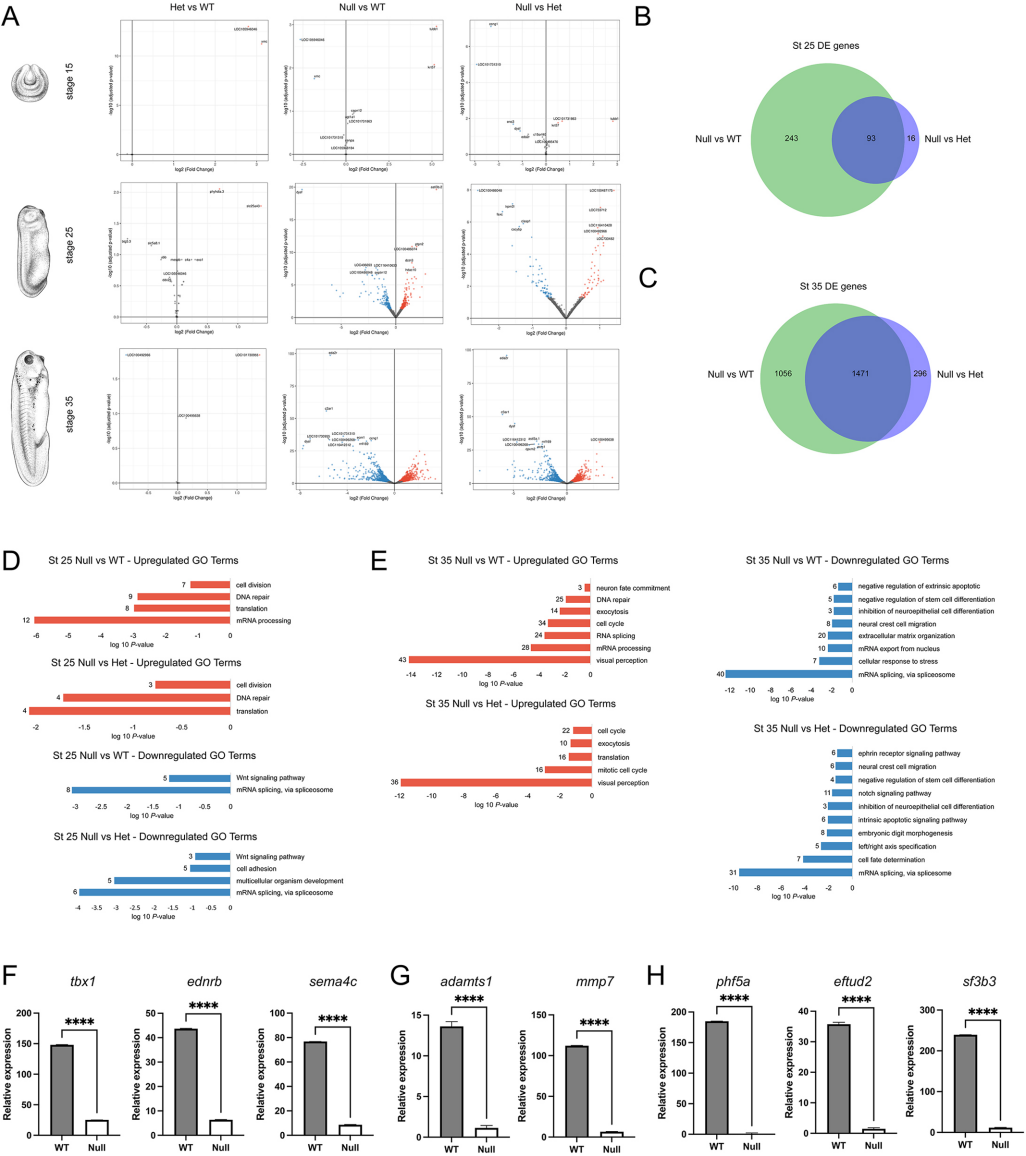
**RNA-seq analysis of differentially expressed genes in WT and *sf3b4* mutant embryos.** (A) Volcano plots showing significance (*y*-axis; log10-adjusted *P*-value) versus amplitude (*x*-axis; log2-fold change) of differentially expressed genes across genotypes and stages. Genotype comparisons (Het versus WT, Null versus WT and Null versus Het) are indicated at the top and embryonic stage (stages 15, 25 and 35) on the left. The top ten genes for each comparison are labeled with their Xenbase ID. *Xenopus* illustrations © Natalya Zahn (Xenbase; www.xenbase.org RRID:SCR_003280) (Zahn et al., 2022). (B,C) Venn diagram analysis of differentially expressed (DE) genes at stage 25 (B) and stage 35 (C), comparing Null versus Wt and Null versus Het. (D,E) Gene Ontology (GO) term analysis of differentially expressed genes at NF stage 25 (D) and stage 35 (E). Upregulated terms are in red, downregulated terms in blue. The numbers at end of the bars indicate the number of genes in each GO category. (F-H) qRT-PCR validation of a subset of downregulated genes in Null versus WT stage 35 embryos, from the ‘neural crest cell migration’ (F), ‘extracellular matrix organization’ (G) and ‘mRNA splicing, via spliceosome’ (H) GO term categories. ****P<0.0001 (Welch's two-tailed unpaired *t*-test). *n*=3. Error bars represent +s.d.

To identify gene networks disrupted in *sf3b4* mutants that may underly the Null phenotype observed, we performed bulk RNA-seq on pools of whole embryos from NF stages 15, 25 and 35 comparing WT, Het and Null at each stage. Principal component analysis (PCA) at all three stages indicated that WT and Het animals are more similar to each other than to the Null (Fig. S5). Differential gene expression analyses indicated very minimal changes at NF stage 15 across genotypes (Fig. 6A, top row). Furthermore, WT and Het samples analyses at all three stages showed little to no difference (Fig. 6A, left column; Table S3), suggesting that loss of one copy of *sf3b4* is largely inconsequential at these stages. By contrast, pairwise comparison of Null versus WT and Null versus Het at NF stage 25 and stage 35 showed a significant number of differentially expressed genes (Fig. 6A, middle and right columns). We next used Venn diagrams to show the extent to which gene expression changes in Null versus WT and Null versus Het samples overlap at these two stages (Fig. 6B,C). Interestingly, the majority of differentially expressed genes in Null versus Het largely overlapped with that of Null versus WT at stage 25 (Fig. 6B) and stage 35 (Fig. 6C), although the overall number of differentially expressed genes was much greater at stage 35 (2823 genes) than at stage 25 (352 genes) (Fig. 6C). It is important to point out that the vast majority of these genes were altered in the same direction (up or down) in the two comparisons, as illustrated for a subset of genes in Table S4.

We used the list of overlapping genes at stage 25 (Fig. 6B; 93 genes) and stage 35 (Fig. 6C; 1471 genes) to perform GO analyses for Biological Processes. This revealed an enrichment for terms such as ‘Wnt signaling pathway’ and ‘mRNA splicing, via spliceosome’ for downregulated genes, and terms such as ‘cell division’ and ‘DNA repair’ for upregulated genes at NF stage 25 (Fig. 6D; Table S5). Whereas at NF stage 35, ‘neural crest cell migration’, ‘extracellular matrix organization’, ‘negative regulation of extrinsic apoptotic signaling’ and ‘mRNA splicing, via spliceosome’ terms were enriched for downregulated genes, and ‘cell cycle’, ‘exocytosis’ and ‘visual perception’ terms enriched for upregulated genes (Fig. 6E; Table S6).

We next performed qRT-PCR to validate a subset of downregulated genes in these categories using RNA isolated from new batches of Null and WT stage 35 embryos. Our results revealed a significant decrease in expression levels of *tbx1*, *ednrb* and *sema4c* as representative genes for the ‘neural crest cell migration’ GO term (Fig. 6F), *adamts1* and *mmp7* as representative genes for the ‘extracellular matrix organization’ GO term (Fig. 6G), and *phf5a*, *eftud2* and *sf3b3* as representative genes for the ‘mRNA splicing, via spliceosome’ GO term (Fig. 6H) in Null compared to WT embryos. Taken together, these results demonstrate that gene expression changes in WT versus Het are very minimal at all stages examined, suggesting a limited impact associated with the loss of one copy of *sf3b4*, whereas when both genotypes were compared to their Null counterpart a similar dysregulation of gene expression was observed, which was significantly more pronounced at stage 35.

### Altered splicing events associated with Sf3b4 loss of function

**Fig. 7.**
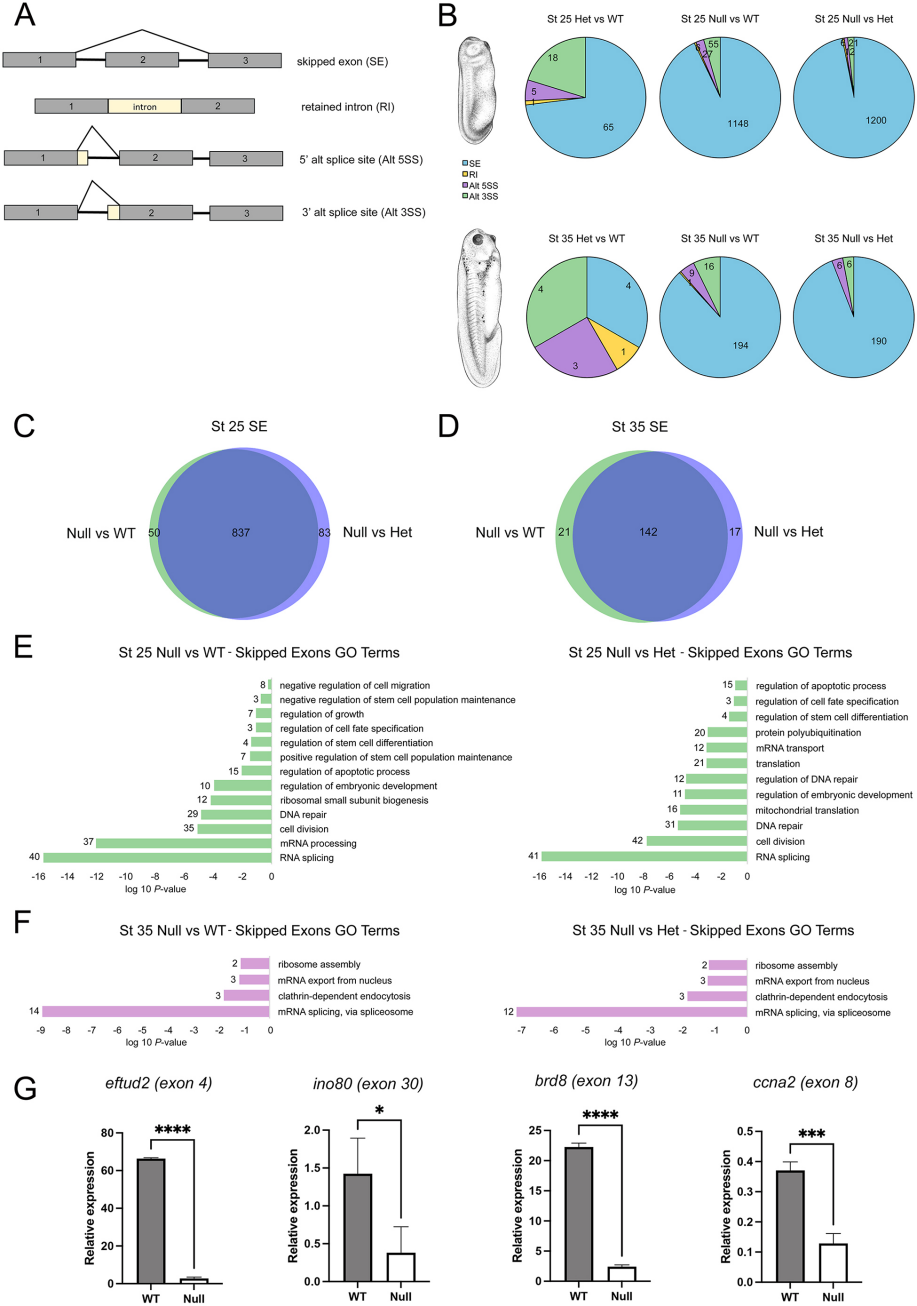
**RNA-seq analysis of splicing events in WT and *sf3b4* mutant embryos.** (A) Diagram of splicing events considered in this analysis. (B) Pie charts showing the type and number of splicing events occurring for each genotype comparison (Het versus WT, Null versus WT and Null versus Het) at stage 25 and stage 35. *Xenopus* illustrations © Natalya Zahn (Xenbase; www.xenbase.org RRID:SCR_003280) (Zahn et al., 2022). (C,D) Venn diagrams for genes with skipped exon at NF stage 25 (C) and stage 35 (D). WT and Het samples show substantial overlap at both stages. (E,F) GO term analysis for genes with skipped exon at NF stage 25 (E) and stage 35 (F). The numbers at end of the bars indicate the number of genes in each GO category. (G) qRT-PCR validation of a subset of representative genes with abnormal skipped exon in Null versus WT stage 25 embryos, from the ‘RNA splicing’ (*eftud2*), ‘regulation of apoptotic process’ (*brd8*), ‘cell division’ (*ccna2*) and ‘regulation of embryonic development’ (*ino80*) GO term categories. *P<0.05, ***P<0.001, ****P<0.0001 (Welch's two-tailed unpaired *t*-test). *n*=3. Error bars represent +s.d.

Because Sf3b4 is an active component of the splicing machinery, we next examined the number and type of splicing events occurring at each stage (NF stages 15, 25 and 35), comparing each genotype, and focusing on four main events: skipped exons, retained introns, and 3′ and 5′ alternative splice sites (Fig. 7A). As observed for differentially expressed genes (Fig. 6A), we found very little difference between WT and Het when compared to Null at all three stages examined (Fig. 7B; Fig. S6). However, in all cases there was a marked increase in the number of genes with abnormal skipped exon events, especially at stage 25 (Fig. 7B). Venn diagram representation highlights the considerable overlap in genes with aberrant skipped exon between WT and Het when compared to Null at stage 25 (Fig. 7C) and stage 35 (Fig. 7D), with a greater overall number of genes affected at stage 25 (970 genes) than at stage 35 (180 genes) or stage 15 (140 genes) (Fig. 7C,D; Fig. S6).

Interestingly, two-thirds of the genes with aberrant skipped exon events at stage 35 also had skipped exon events at stage 25 (106 out of 163 in WT compared to Null and 106 out of 159 in Het compared to Null), indicating a consistent requirement for Sf3b4 splicing activity (Fig. S7). Genes that show aberrant skipped exon events at stage 25 but not stage 35 may be expressed at low level or not at all at later stages; this is, for example, the case for *chd2*. This gene has an aberrant skipped exon event at stage 25, but not stage 35 when *chd2* expression levels are much lower (Fisher et al., 2023).

We used the list of overlapping genes at stage 25 (Fig. 7C; 837 genes) and stage 35 (Fig. 7D; 142 genes) to perform GO analyses for Biological Processes. The analysis at stage 25 revealed an enrichment for terms such as ‘RNA splicing’, ‘DNA repair’, ‘regulation of embryonic development’ and ‘regulation of apoptotic process’, among others (Fig. 7E; Table S7), and at stage 35 these terms included ‘mRNA splicing, via spliceosome’, ‘clathrin-dependent endocytosis’, ‘mRNA export from nucleus’ and ‘ribosome assembly’ (Fig. 7F; Table S8). We next performed qRT-PCR to validate a subset of genes displaying abnormal skipped-exon events from these categories using RNA isolated from new batches of Null and WT stage 25 embryos. Our results confirmed that *eftud2* (RNA splicing), *ino80* (regulation of embryonic development), *brd8* (regulation of apoptotic process) and *ccna2* (cell division) show aberrant skipped exons in Null compared to WT (Fig. 7G). These results indicate that the complete loss of Sf3b4 function affects the spliceosome activity by preferentially promoting atypical skipped-exon events more widely at stage 25 than at stage 35, whereas the loss of one copy of *sf3b4* had limited impact on spliceosome activity at both stages.

## DISCUSSION

Here, we report the generation and molecular characterization of a *Xenopus tropicalis* CRISPR/Cas9 *sf3b4* mutant line, as a novel tool to investigate the pathogenesis of Nager syndrome, a condition that affects NC-derived craniofacial structures*.* We show that in the absence of Sf3b4 function NC induction is not affected. However, NC cell migration is disrupted at the tailbud stage, and coupled with an increase in apoptosis in the head region, a phenotype that is much more pronounced in Null than in Het animals. *sf3b4* Null animals failed to survive beyond the tadpole stage, but Het tadpoles were indistinguishable from their WT siblings, forming largely normal craniofacial cartilages. Temporal RNA-seq analysis confirmed at the transcriptome level the limited difference between WT and *sf3b4* Het embryos, and revealed aberrant pre-mRNA splicing events in stage 25 Null embryos, most notably characterized by an increase in the number of genes with atypical skipped exons, followed by a massive dysregulation of gene expression at stage 35. GO analysis of differentially expressed genes in *sf3b4* Null embryos revealed an enrichment in terms pertaining to mRNA splicing, apoptosis, cell cycle and NC cell migration. Altogether, our data indicate that *sf3b4* haploinsufficiency is compatible with normal development in *X. tropicalis*, whereas homozygous deletion of *sf3b4* is detrimental to survival and the formation of NC-derived cartilage progenitors, consistent with an autosomal recessive mode of inheritance. We also found that the *sf3b4* Null embryos exhibit heart defects as evidenced by the reduced expression of several cardiac genes at stage 35, and an enrichment for GO terms related to cardiac morphogenesis in the transcriptomic analysis of Het versus Null embryos.

Splicing factor variants have been implicated in numerous diseases, referred to as spliceosomopathies. They form a singular group of diseases due to the nature of their phenotype affecting often a single cell or tissue type, despite the predicted ubiquitous activity of the spliceosome in all cell types of the body (Griffin and Saint-Jeannet, 2020; Beauchamp et al., 2020). The tissues primarily affected in these pathologies include the retina (retinitis pigmentosa), spinal cord (amyotrophic lateral sclerosis), bone marrow (myelodysplastic syndromes), limb and the craniofacial skeleton (Griffin and Saint-Jeannet, 2020). Understanding the cell/tissue specificity of these phenotypes has been challenging.

Mouse embryos with heterozygous *Sf3b4* mutation show axial skeleton and forebrain defects with no apparent craniofacial abnormalities, whereas *Sf3b4* homozygous deletion is embryonic lethal (Yamada et al., 2020; Kumar et al., 2023). We also report that *X. tropicalis sf3b4* homozygous deletion is not compatible with survival, although these animals survive early on, presumably due to a maternal supply of Sf3b4 mRNA or protein (Fisher et al., 2023). Similarly to the mouse phenotype, *X. tropicalis sf3b4* heterozygous animals do not show any obvious craniofacial anomalies. Whereas in humans *SF3B4* haploinsufficiency causes severe craniofacial abnormalities, the lack of an overt craniofacial phenotype in heterozygous mouse and frog embryos could be explained by the activity of other genes that compensate for the loss of *sf3b4*.

Interestingly, we also detected a heart defect in null embryos at later stages of development, which we posit contributes to the poor survival of the nulls at tadpole stages. The mechanisms underlying this phenotype remain unclear, and will require further investigation. Although cardiac neural crest cells participate minimally to the *Xenopus* heart, primarily contributing to the aortic sac and arch arteries (Lee and Saint-Jeannet, 2011), we speculate that cardiac NC cell migration and survival is also affected in the null mutants. Individuals with Nager syndrome do not typically present with cardiac defects; however, individuals with Rodríguez syndrome have cardiac abnormalities, including atrial and ventricular septal defects (Rodríguez et al., 1990).

RNA-seq analyses of mouse *Sf3b4* heterozygous mutants reveal a disruption in Hox gene expression and aberrant splicing of several chromatin remodelers known to regulate Hox genes (Kumar et al., 2023). We also found downregulation of the Hox genes *hoxa1* and *hoxb6* in our RNA-seq data, as well as aberrant splicing of several chromatin remodelers, including *kmt2e*, *kmt2a*, *phf20*, *phf10*, *scmh1*, *chd7*, *chd6* and *chd2*. The same group recently generated a conditional deletion of *Sf3b4* in the NC lineage using the *Wnt1-Cre2* transgenic line (Kumar et al., 2024). These animals exhibit craniofacial and cardiac defects with variable penetrance and expressivity. The authors propose that the levels of Sf3b4 in non-NC derived neighboring tissues directly influence the severity of the phenotype. At later stages, both NC- and mesoderm-derived craniofacial bones and cartilages were hypoplastic in the mutants, together with abnormal cranial ganglia and cardiac outflow tract defects, presumably due to changes in gene expression related to NC development, cell survival and proliferation (Kumar et al., 2024).

In *X. laevis*, morpholino-mediated knockdown of *sf3b4* results in a reduction of NC gene expression at neurula and tailbud stages, causing a reduction of NC-derived craniofacial cartilages at tadpole stages through a mechanism that involves apoptosis (Devotta et al., 2016). We did not observe a similar reduction in NC gene expression in the *sf3b4 X. tropicalis* mutant embryos. Although the *X. laevis* morphant phenotype was partially rescued by expression of human SF3B4 (Devotta et al., 2016), we cannot exclude the possibility that the morpholino may still also have off-target effects that could explain the observed reduced expression of premigratory NC genes. There is also the possibility that compensatory mechanisms may be at play in the CRISPR/Cas9-induced *sf3b4* mutation, with a subset of genes compensating at early stages for the loss of *sf3b4*, a phenomenon that is not seen to the same extent in the context of morpholino knockdowns (Rossi et al., 2015). Regardless, in both models, cell death appears to be a common root cause in the presentation of the phenotype. Although *X. tropicalis sf3b4* Het embryos exhibit a mild NC phenotype at tailbud stages, they appear to regulate/compensate this defect as they show virtually no craniofacial differences with WT animals at tadpole stage. Our data indicate that loss of Sf3b4 in *X. tropicalis* manifests differently than in humans. The *sf3b4* Het embryos do not show an overall robust disease phenotype, and in many respects are much more similar to WT than Null embryos, suggesting that redundant mechanisms are at play in *Xenopus*. This is further supported by our RNA-seq data (see below). Although this tool does not fully recapitulate the human disease condition, it is an excellent system to investigate the molecular processes regulated by Sf3b4 during NC and craniofacial development.

Our RNA-seq analyses at three different stages of NC development – pre-migratory (NF stage 15), migratory (NF stage 25) and post-migratory (NF stage 35) – indicate that there is very little change in gene expression between WT and *sf3b4* Het animals (Fig. 6A), suggesting that loss of one copy of *sf3b4* is largely inconsequential in *X. tropicalis*, which is consistent with the phenotypes of these embryos (Fig. 5). When both genotypes were individually compared to *sf3b4* Null, we observed a similar pattern of gene dysregulation, with a much greater number of genes affected at stage 35 than at stage 25 or stage 15 (Fig. 6B,C; Fig. S6). GO analyses of these gene sets revealed terms such as ‘mRNA splicing, via spliceosome’, ‘apoptosis’ and ‘neural crest cell migration’ enriched in genes downregulated at stage 35, whereas the most statistically significant GO term enriched among upregulated genes was ‘visual perception’. Genes downregulated related to ‘mRNA splicing, via spliceosome’ include several genes that have been linked to other craniofacial spliceosomopathies, such as *eftud2* (Lines et al., 2012), *txnl4* (Wieczorek et al., 2014) and *phf5a* (Harms et al., 2023). We also found multiple genes causative of retinitis pigmentosa (a retina-specific spliceosomopathy, characterized by degeneration of photoreceptors), including *prpf3*, *prpf8* and *prpf31* (Tanackovic et al., 2011), and *snrnp200* (Zhao et al., 2009). This is consistent with a recent study describing a photoreceptor phenotype in *sf3b4* mutant zebrafish larvae (Ulhaq et al., 2023). Also downregulated were genes related to ‘apoptosis’, including *cdkn1a* (cyclin dependent kinase inhibitor 1A), a negative regulator of p53 (El-Deiry et al., 1994) and *tp53* (tumor protein 53) itself (Chipuk et al., 2003). This is significant, as previous work has implicated apoptosis in craniofacial malformations underlying loss of Eftud2, Snrpb and Txnl4a in *X. laevis* (Park et al., 2022), as well as the specific involvement of the p53 pathway in *Eftud2* mutant mice (Beauchamp et al., 2021), indicating a common mechanism underlying craniofacial spliceosomopathies. Under the ‘neural crest cell migration’ GO term, we recovered several members of the semaphorin family (*sema3b*, *sema4c*, *sema6a* and *sema6c*), a semaphorin receptor (*nrp1*; neuropilin 1), *ednrb* (endothelin receptor type B) and two transcription factors, *hif1a* and *tbx1*. Most of these factors have been linked to NC development in several species, and pathogenetic variants of *TBX1* and *EDNRB* cause two neurocristopathies, DiGeorge syndrome (Merscher et al., 2001) and Hirschsprung disease (Amiel et al., 1996), respectively. Future studies will determine whether the downregulation of these genes in *sf3b4* Null mutants underlies aspects of the NC-specific phenotype of this craniofacial spliceosomopathy.

The RNA-seq analyses combined with rMATS, a computational tool to detect splicing events, revealed very limited changes in the number and type of splicing events occurring between WT and *sf3b4* Het animals (Fig. 7B), again suggesting that one copy of *sf3b4* in frogs is sufficient for spliceosome activity. When both genotypes are individually compared to *sf3b4* Null samples, a similar pattern of disrupted splicing events emerges, with a strong bias toward skipped exons, and a much greater number of genes affected at stage 25 than at stage 35 (Fig. 7C,D). This bias toward skipped exons could be due to the role of Sf3b4 in tethering the U2 complex to the branch site during splicing (Champion-Arnaud et al., 1994). In the absence of Sf3b4, the branch site stability may decrease, leading to the spliceosome falling off and exons skipping as alternate branch points are used instead. Interestingly, however, an enrichment in skipped-exon events has been reported for mutation in other components of the spliceosome, including Eftud2 (Beauchamp et al., 2021) and Snrpb (Alam et al., 2022), which carry very different functions in the spliceosome.

GO analyses identified several terms pertaining to ‘mRNA splicing’, ‘regulation of apoptotic process’, ‘DNA repair’, ‘cell division’ and ‘regulation of embryonic development’ were enriched at stage 25, and at stage 35 the most statistically significant GO term enriched was ‘mRNA splicing, via spliceosome’, followed by ‘clathrin dependent endocytosis’. How these factors relate to NC and craniofacial development remain to be investigated. There is very little overlap between the genes that are dysregulated and the genes that are aberrantly spliced in our dataset. This could be explained by the fact that genes that are aberrantly spliced are still expressed at WT levels, but the expression of their downstream effectors are up- or downregulated due to these changes in splicing events. This question remains open as these mechanistic details require further elucidation.

The temporal transcriptomic analysis described here offers a unique perspective on the sequence of events and the mechanisms of Sf3b4 pathogenesis. *sf3b4* mutants show a massive disruption in spliceosome activity occurring primarily between NF stage 15 and stage 25, followed by a significant dysregulation of gene expression occurring around NF stage 35. This suggests that changes in gene expression are the likely consequence of spliceosome activity disruption and therefore may not be the primary cause of the phenotype, but rather the contributing factor. Future studies will focus on the plethora of genes misregulated in Null mutants at stage 35 (Table S6) to identify the gene networks and pathways underlying this craniofacial condition.

## MATERIALS AND METHODS

### Animal care

*X. tropicalis* were obtained from The National *Xenopus* Resource (NXR; Woods Hole, MA, USA) housed as described (McNamara et al., 2018; Shaidani et al., 2020). Adult *sf3b4* −31/+ mutants were intercrossed by *in vitro* fertilization to produce embryos for this study. Females were given 20 U of pregnant mare serum gonadotropin (BioVender, RP17827210000) and 200 U of human chorionic gonadotropin (BioVender, RP17825010) to induce egg laying (Wlizla et al., 2018).

### WMISH

Antisense digoxygenin (DIG)-labeled probes (Genius kit, Roche) were synthesized using template cDNA encoding *X. tropicalis sf3b4* (Horizon, MXT1765-202789577) and *X. laevis sox10* (Aoki et al., 2003), *snail2* (Mayor et al., 1995), *tfap2e* (Hong et al., 2014), *twist1* (Hopwood et al., 1989), *sox9* (Spokony et al., 2002), *runx2* (Kerney et al., 2007), *tbx5* (Brown et al., 2005), *tbx20* (Brown et al., 2002) and *nkx2-5* (Cleaver et al., 1996). WMISH was performed as previously described (Harland, 1991). For double *in situ* hybridization, *sf3b4*-DIG- and *sox10*-FITC-labeled RNA probes were sequentially detected using FITC- and DIG alkaline phosphatase-conjugated antibodies. *sox10* was visualized using Magenta Phosphate (Biosynth) and the color reaction to detect *sf3b4* was performed using 5-bromo-4-chloro-3-indolyl-phosphate (Roche), as described (Saint-Jeannet, 2017).

### CRISPR/Cas9

Guide RNAs (sgRNAs) were designed utilizing CRISPRScan (https://www.crisprscan.org/) targeting the first three exons of *sf3b4* (Moreno-Mateos et al., 2015): T1: GGTGCCACGGTGTATGTCGG, T2: GGAACATGATAAAGCTCTAT, T3: GGGGTCTCTCATGATCTTGG, T4: GGCTTCGGACGCAGCCATTG and T5: GGGGCGAGAAAATGGCGGCT. 5′ dinucleotides were converted to GG for increased mutagenic activity (Gagnon et al., 2014). The SP6 MEGAscript kit (Ambion, AM1330) protocol was followed for synthesis of all sgRNAs. Injections using the *X. tropicalis* Nigerian line (RRID: NXR_1018) at the one-cell stage consisted of 500 pg of guide RNA and 1000 pg of Cas9 protein into each embryo. One F0 female was outcrossed by *in vitro* fertilization to a WT male, resulting in F1 embryos with −5 bp or −31 bp mutations. The −31/+ (*Xtr.sf3b4^emNXR^*; RRID: NXR_3056) *sf3b4* mutant line is available through the NXR (https://www.mbl.edu/xenopus).

### Embryo collection and genotyping of tissue samples

To genotype adult frogs, tissue samples were taken from webbing on hindlimbs using single-use biopsy punches (VWR, 21909-140). Heterozygous mating pairs were intercrossed to produce embryos for *in situ* hybridization and RNA-seq analyses. Embryos collected for *in situ* hybridization were fixed in MEMFA (10 ml 10× MEMFA salts, 10 ml 37% formaldehyde, 80 ml nanofiltrated H_2_O) overnight at 4°C. Embryos were stored long term at −20°C in 100% ethanol. Ventral tissue (stages 15-20) and tail clips (stages 25-45) for genomic DNA (gDNA) extraction were taken from fixed embryos after rehydration in PBS. For embryos used in RNA-seq analysis, the ventral (stage 15) and posterior (stage 25 and stage 35) parts of the embryos were dissected and collected for genotyping, and the remaining tissues were immediately preserved on dry ice and stored at −80°C.

gDNA extractions were performed using the QIAGEN DNeasy Blood & Tissue Kit (69506). PCR amplification was carried out using the following primers for the targeted region: forward primer 5′-AATGAAACACCCTCTATGCGC-3′ and reverse primer 5′-AGAGATGGAGCCTGCACC-3′. The PCR product was then purified by following the NucleoSpin PCR Clean-up procedure (Macherey-Nagel; 740609.250) and sent to Genewiz, South Plainfield, NJ, USA and The Keck Facility, Woods Hole, MA, USA for Sanger sequencing to confirm genotypes.

### Western blot analysis

Pools of ten embryos were homogenized in lysis buffer and concentrated, and western blot analysis was performed as previously described (Devotta et al., 2016). Primary antibodies were: anti-Sf3b4 polyclonal antibody (Proteintech, 10482-1-AP; 1:2000) and anti-α-tubulin antibody (Sigma-Aldrich, T9026; 1:500). Secondary antibodies were: donkey anti-rabbit (EMD Millipore, MAB201P) and donkey anti-mouse IgG (Abcam, ab6820) coupled to horseradish peroxidase (1:10,000).

### Quantification of head depth and length

Using ImageJ software, a vertical line was drawn behind the eye to define head depth, and a horizontal line extending from the back of the eye to the front of the face from the eye to define head length (Fig. 2E, top embryos).

### qRT-PCR analysis

Total RNA was extracted from embryos using the RNeasy microRNA isolation kit (QIAGEN) and the RNA samples were digested with RNase-free DNase I to eliminate genomic DNA. RT-qPCR analysis was performed using the Power SYBR™ Green RNA to C_T_™ 1 step RT-PCR kit (Applied Biosystems, 4389986) on a QuantStudio 3 Real-Time PCR System (Applied Biosystems) using gene-specific primer sets (Table S9).

### Quantification of *in situ* hybridization signal

The *in situ* hybridization signal was measured using ImageJ software. The image was reverted to an 8-bit gray image and inverted. The area of staining was selected, and the mean gray scale was calculated for each area. The left and right sides of each embryo were calculated separately and then averaged together for the final value. Two-tailed Welch's unpaired *t*-test was performed using Prism GraphPad (v.10.01) to determine statistical significance.

### Quantification of migratory streams

The length of NC streams was measured using ImageJ software by drawing a vertical line between the dorsal midline and the ventral-most aspect of the *sox10* and *sox9* expression domains in each stream (streams 2, 3 and 4). The ratio of stream length to total embryo depth (dorsal-ventral axis) was then calculated. Two-tailed Welch's unpaired *t*-test was performed using Prism GraphPad (v.10.0.1) to determine statistical significance.

### TUNEL assay and quantification

TUNEL staining was carried out as previously described (Hensey and Gautier, 1998; Devotta et al., 2016). To quantify cell death, embryos were imaged individually, and a set region of the head encompassing the branchial arches and excluding the eye was chosen in which the number of TUNEL-positive cells were manually counted for each embryo. Two-tailed Welch's unpaired *t*-test was performed using Prism GraphPad (v.10.0.1) to determine statistical significance.

### Cartilage staining

Tadpoles at NF stage 45 were fixed in MEMFA overnight at room temperature, rinsed in tap water, skinned and eviscerated. Dissected heads were then stained in Alcian Blue solution (0.06% in 30% acetic acid and 70% ethanol) for 24 h, rinsed in 95% ethanol for 2 h, rehydrated and macerated in 2% potassium hydroxide overnight at room temperature. Specimens were then cleared in increasing concentrations of glycerol (20%, 40%, 60% and 80%) in 2% potassium hydroxide and imaged on a Leica M165 stereomicroscope (Leica Microsystems Inc.).

### RNA isolation and sequencing

Post-genotyping, pools of five embryos were used for RNA extraction using the QIAGEN RNeasy Micro Kit (74004). RNA was eluted in RNase-free water and sent to the New York University Genome Technology Center for RNA sequencing. RNA QC/QA was performed on a bioanalyzer before performing automated stranded RNA-seq library preparation with polyA selection. Samples were run on the Illumina NovaSeq 6000 system with SP 100 cycle flow cells.

### RNA-seq data analysis

RNA-seq data were analyzed using the sns rna-star pipeline (https:igordot.github.io/sns/routes/rna-star.html). Adapters and low-quality bases were trimmed using Trimmomatic (v.0.36) (Bolger et al., 2014). Sequencing reads were mapped to the reference genome (XENTR_10.0; https://download.xenbase.org/xenbase/Genomics/JGI/Xentr10.0) using the STAR aligner (v.2.7.3) (Dobin et al., 2013). Alignments were guided by a Gene Transfer Format (GTF) file, which was converted from a General Feature Format (GFF3) file. The mean read insert sizes and their standard deviations (+s.d.) were calculated using Picard tools (v.2.18.20) (http://broadinstitute.github.io/picard). The genes-samples counts matrix was generated using featureCounts (v.1.6.3) (Liao et al., 2014), normalized based on their library size factors using DEseq2 (v.1.30.1) (Love et al., 2014), and differential expression analysis was performed. The read per million normalized BigWig files were generated using deepTools (v.3.1.0) (Ramirez et al., 2016). To compare the level of similarity among the samples and their replicates, we used two methods: PCA and Euclidean distance-based sample clustering. All the downstream statistical analyses and generating plots were performed in R environment (v.4.0.3) (https://www.r-project.org/). Differentially expressed genes were determined with a cutoff of less than −0.4 or more than 0.4 log2FC.

rMATS (v.4.0.2) (Shen et al., 2014) was used for detecting differential alternative splicing events from RNA-seq data. Events with a mean of inclusion junction counts or a mean of skipped junction counts <10 were removed. Only events with an inclusion level difference of more than 0.1 or less than −0.1, with *P*<0.05 were included.

The results of GO analysis were generated by DAVID 2021 (Huang et al., 2007) using gene lists from differential expressed genes and splicing events with conversion to human terms. Non-conserved terms were removed from analysis. Venn diagrams were generated using DeepVenn (Hulsen et al., 2022 preprint).

## Supplementary Material

10.1242/dmm.052169_sup1 Supplementary information

Table S2. GO analysis of downregulated genes in *sf3b4* mutants at stage 35 shows an enrichment for cardiac terms.

Table S3. Differentially expressed genes in WT vs Het at stage 25.

Table S4. GO analysis of differentially expressed genes in *sf3b4* mutants at stage 25.

Table S5. GO analysis of differentially expressed genes in *sf3b4* mutants at stage 35.

Table S6. GO analysis of genes with aberrant skipped exons in *sf3b4* mutants at stage 25.

Table S7. GO analysis of genes with aberrant skipped exons in *sf3b4* mutants at stage 35.

Table S8. Comparison of genes differentially expressed in Null vs WT and Null vs Het for a sample of 20 genes at stage 25 and stage 35.

## References

[DMM052169C1] Alam, S. S., Kumar, S., Beauchamp, M.-C., Bareke, E., Boucher, A., Nzirorera, N., Dong, Y., Padilla, R., Zhang, S. J., Majewski, J. et al. (2022). Snrpb is required in murine neural crest cells for proper splicing and craniofacial morphogenesis. *Dis. Model. Mech.* 15, dmm049544. 10.1242/dmm.049544 35593225 PMC9235875

[DMM052169C2] Amiel, J., Attie, T., Jan, D., Pelet, A., Edery, P., Bidaud, C., Lacombe, D., Tam, P., Simeoni, J., Flori, E. et al. (1996). Heterozygous endothelin receptor B (EDNRB) mutations in isolated Hirschsprung disease. *Hum. Mol. Genet.* 5, 355-357. 10.1093/hmg/5.3.355 8852660

[DMM052169C3] Aoki, Y., Saint-Germain, N., Gyda, M., Magner-Fink, E., Lee, Y.-H., Credidio, C. and Saint-Jeannet, J.-P. (2003). Sox10 regulates the development of neural crest-derived melanocytes in Xenopus. *Dev. Biol.* 259, 19-33. 10.1016/S0012-1606(03)00161-1 12812785

[DMM052169C4] Beauchamp, M. C., Alam, S. S., Kumar, S. and Jerome-Majewska, L. A. (2020). Spliceosomopathies and neurocristopathies: two sides of the same coin? *Dev. Dyn.* 249, 924-945. 10.1002/dvdy.183 32315467

[DMM052169C5] Beauchamp, M.-C., Djedid, A., Bareke, E., Merkuri, F., Aber, R., Tam, A. S., Lines, M. A., Boycott, K. M., Stirling, P. C., Fish, J. L. et al. (2021). Mutation in *Eftud2* causes craniofacial defects in mice via mis-splicing of *Mdm2* and increased P53. *Hum. Mol. Genet.* 30, 739-757. 10.1093/hmg/ddab051 33601405 PMC8161524

[DMM052169C6] Bernier, F. P., Caluseriu, O., Ng, S., Schwartzentruber, J., Buckingham, K. J., Innes, A. M., Jabs, E. W., Innis, J. W., Schuette, J. L., Gorski, J. L. et al. (2012). Haploinsufficiency of SF3B4, a component of the pre-mRNA spliceosomal complex, causes Nager syndrome. *Am. J. Hum. Genet.* 90, 925-933. 10.1016/j.ajhg.2012.04.004 22541558 PMC3376638

[DMM052169C7] Bolger, A. M., Lohse, M. and Usadel, B. (2014). Trimmomatic: a flexible trimmer for Illumina sequence data. *Bioinformatics* 30, 2114-2120. 10.1093/bioinformatics/btu170 24695404 PMC4103590

[DMM052169C8] Brown, D. D., Binder, O., Pagratis, M., Parr, B. A. and Conlon, F. L. (2002). Developmental expression of the Xenopus laevis Tbx20 orthologue. *Dev. Genes Evol.* 212, 604-607. 10.1007/s00427-002-0276-6 12536325 PMC1635808

[DMM052169C9] Brown, D. D., Martz, S. N., Binder, O., Goetz, S. C., Price, B. M. J., Smith, J. C. and Conlon, F. L. (2005). Tbx5 and Tbx20 act synergistically to control vertebrate heart morphogenesis. *Development* 132, 553-563. 10.1242/dev.01596 15634698 PMC1635804

[DMM052169C10] Champion-Arnaud, P. and Reed, R. (1994). The prespliceosome components SAP 49 and SAP 145 interact in a complex implicated in tethering U2 snRNP to the branch site. *Genes Dev.* 8, 1974-1983. 10.1101/gad.8.16.1974 7958871

[DMM052169C11] Chemke, J., Mogilner, B. M., Ben-Itzhak, I., Zurkowski, L. and Ophir, D. (1988). Autosomal recessive inheritance of Nager acrofacial dysostosis. *J. Med. Genet.* 25, 230-232. 10.1136/jmg.25.4.230 3367347 PMC1015502

[DMM052169C12] Chipuk, J. E., Maurer, U., Green, D. R. and Schuler, M. (2003). Pharmacologic activation of p53 elicits Bax-dependent apoptosis in the absence of transcription. *Cancer Cell* 4, 371-381. 10.1016/S1535-6108(03)00272-1 14667504

[DMM052169C13] Cleaver, O. B., Patterson, K. D. and Krieg, P. A. (1996). Overexpression of the tinman-related genes XNkx-2.5 and XNkx-2.3 in Xenopus embryos results in myocardial hyperplasia. *Development* 122, 3549-3556. 10.1242/dev.122.11.3549 8951070

[DMM052169C14] Czeschik, J. C., Voigt, C., Alanay, Y., Albrecht, B., Avci, S., Fitzpatrick, D., Goudie, D. R., Hehr, U., Hoogeboom, A. J., Kayserili, H. et al. (2013). Clinical and mutation data in 12 patients with the clinical diagnosis of Nager syndrome. *Hum. Genet.* 132, 885-898. 10.1007/s00439-013-1295-2 23568615

[DMM052169C15] Devotta, A., Juraver-Geslin, H., Gonzalez, J. A., Hong, C.-S. and Saint-Jeannet, J.-P. (2016). Sf3b4-depleted Xenopus embryos: a model to study the pathogenesis of craniofacial defects in Nager syndrome. *Dev. Biol.* 415, 371-382. 10.1016/j.ydbio.2016.02.010 26874011 PMC4914463

[DMM052169C16] Dobin, A., Davis, C. A., Schlesinger, F., Drenkow, J., Zaleski, C., Jha, S., Batut, P., Chaisson, M. and Gingeras, T. R. (2013). STAR: ultrafast universal RNA-seq aligner. *Bioinformatics* 29, 15-21. 10.1093/bioinformatics/bts635 23104886 PMC3530905

[DMM052169C17] Drivas, T. G., Taylor, J. A. and Zackai, E. H. (2019). The final demise of Rodriguez lethal acrofacial dysostosis: a case report and review of the literature. *Amer. J. Med. Genet.* 179, 1063-1068. 10.1002/ajmg.a.61121 30924273

[DMM052169C18] El-Deiry, W. S., Harper, J. W., O'Connor, P. M., Velculescu, V. E., Canman, C. E., Jackman, J., Pientenpol, J. A., Burrell, M., Hill, D. E., Wang, Y. et al. (1994). WAF1/CIP1 is induced in p53-mediated G1 arrest and apoptosis. *Cancer Res.* 54, 1169-1174. 8118801

[DMM052169C19] Fisher, M., James-Zorn, C., Ponferrada, V., Bell, A. J., Sundararaj, N., Segerdell, E., Chaturvedi, P., Bayyari, N., Chu, S., Pells, T. et al. (2023). Xenbase: key features and resources of the Xenopus model organism knowledgebase. *Genetics* 224, iyad018. 10.1093/genetics/iyad018 36755307 PMC10158840

[DMM052169C20] Gagnon, J. A., Valen, E., Thyme, S. B., Huang, P., Ahkmetova, L., Pauli, A., Montague, T. G., Zimmerman, S., Richter, C. and Schier, A. F. (2014). Efficient mutagenesis by Cas9 protein-mediated oligonucleotide insertion and large-scale assessment of single-guide RNAs. *PLoS ONE* 9, e98186. 10.1371/journal.pone.0098186 24873830 PMC4038517

[DMM052169C21] Griffin, C. and Saint-Jeannet, J.-P. (2020). Spliceosomopathies: diseases and mechanisms. *Dev. Dyn.* 249, 1038-1046. 10.1002/dvdy.214 32506634 PMC8603363

[DMM052169C22] Halal, F., Herrmann, J., Pallister, P. D., Opitz, J. M., Desgranges, M. F. and Grenier, G. (1983). Differential diagnosis of Nager acrofacial dysostosis syndrome: report of four patients with Nager syndrome and discussion of other related syndromes. *Am. J. Med. Genet.* 14, 209-224. 10.1002/ajmg.1320140203 6837625

[DMM052169C23] Hall, B. D. (1989). Nager acrofacial dysostosis: autosomal dominant inheritance in mild to moderately affected mother and lethally affected phocomelic son. *Am. J. Med. Genet.* 33, 394-397. 10.1002/ajmg.1320330321 2801774

[DMM052169C24] Harland, R. M. (1991). In situ hybridization: an improved whole-mount method for Xenopus embryos. *Methods Cell Biol.* 36, 685-695. 10.1016/S0091-679X(08)60307-6 1811161

[DMM052169C25] Harms, F. L., Dingemans, A. J. M., Hempel, M., Pfundt, R., Bierhals, T., Casar, C., Müller, C., Niermeijer, J.-M. F., Fischer, J., Jahn, A. et al. (2023). De novo PHF5A variants are associated with craniofacial abnormalities, developmental delay, and hypospadias. *Genet. Med.* 25, 100964. 10.1016/j.gim.2023.100964 37728613

[DMM052169C26] Hensey, C. and Gautier, J. (1998). Programmed cell death during Xenopus development: a spatio-temporal analysis. *Dev. Biol.* 203, 36-48. 10.1006/dbio.1998.9028 9806771

[DMM052169C27] Hong, C. S., Devotta, A., Lee, Y. H., Park, B. Y. and Saint-Jeannet, J.-P. (2014). Transcription factor AP2 epsilon (Tfap2e) regulates neural crest specification in Xenopus. *Dev. Neurobiol.* 74, 894-906. 10.1002/dneu.22173 24616412 PMC4107115

[DMM052169C28] Hopwood, N. D., Pluck, A. and Gurdon, J. B. (1989). A Xenopus mRNA related to Drosophila twist is expressed in response to induction in the mesoderm and the neural crest. *Cell* 59, 893-903. 10.1016/0092-8674(89)90612-0 2590945

[DMM052169C29] Huang, D. W., Sherman, B. T., Tan, Q., Collins, J. R., Alvord, W. G., Roayaei, J., Stephens, R., Baseler, M. W., Lane, H. C. and Lempicki, R. A. (2007). The DAVID gene functional classification tool: a novel biological module-centric algorithm to functionally analyze large gene lists. *Genome Biol.* 8, R183. 10.1186/gb-2007-8-9-r183 17784955 PMC2375021

[DMM052169C30] Hulsen, T. (2022). DeepVenn – a web application for the creation of area-proportional Venn diagrams using the deep learning framework Tensorflow.js. *arXiv*, 10.48550/arXiv.2210.04597

[DMM052169C31] Kerney, R., Gross, J. B. and Hanken, J. (2007). Runx2 is essential for larval hyobranchial cartilage formation in Xenopus laevis. *Dev. Dyn.* 236, 1650-1662. 10.1002/dvdy.21175 17474117

[DMM052169C32] Kumar, S., Alam, S. S., Bareke, E., Beauchamp, M.-C., Dong, Y., Chan, W., Majewski, J. and Jerome-Majewska, L. A. (2023). Sf3b4 regulates chromatin remodeler splicing and Hox expression. *Differentiation* 131, 59-73. 10.1016/j.diff.2023.04.004 37167859

[DMM052169C33] Kumar, S., Bareke, E., Lee, J., Carlson, E., Merkuri, F., Schwager, E. E., Maglio, S., Fish, J. L., Majewski, J. and Jerome-Majewska, L. A. (2024). Etiology of craniofacial and cardiac malformations in a mouse model of SF3B4-related syndromes. *Proc. Natl. Acad. Sci. USA* 121, e2405523121. 10.1073/pnas.2405523121 39292749 PMC11441570

[DMM052169C34] Lee, Y.-H. and Saint-Jeannet, J.-P. (2011). Cardiac neural crest is dispensable for outflow tract septation in Xenopus. *Development* 138, 2025-2034. 10.1242/dev.061614 21490068 PMC3082305

[DMM052169C35] Liao, Y., Smyth, G. K. and Shi, W. (2014). FeatureCounts: an efficient general purpose program for assigning sequence reads to genomic features. *Bioinformatics* 30, 923-930. 10.1093/bioinformatics/btt656 24227677

[DMM052169C36] Lines, M. A., Huang, L., Schwartzentruber, J., Douglas, S. L., Lynch, D. C., Beaulieu, C., Guion-Almeida, M. L., Zechi-Ceide, R. M., Gener, B., Gillessen-Kaesbach, G. et al. (2012). Haploinsufficiency of a spliceosomal GTPase encoded by EFTUD2 causes mandibulofacial dysostosis with microcephaly. *Am. J. Hum. Genet.* 90, 369-377. 10.1016/j.ajhg.2011.12.023 22305528 PMC3276671

[DMM052169C37] Love, M. I., Huber, W. and Anders, S. (2014). Moderated estimation of fold change and dispersion for RNA-seq data with DESeq2. *Genome Biol.* 15, 550. 10.1186/s13059-014-0550-8 25516281 PMC4302049

[DMM052169C38] Mayor, R., Morgan, R. and Sargent, M. G. (1995). Induction of the prospective neural crest of Xenopus. *Development* 121, 767-777. 10.1242/dev.121.3.767 7720581

[DMM052169C39] McNamara, S., Wlizla, M. and Horb, M. E. (2018). Husbandry, general care, and transportation of Xenopus laevis and Xenopus tropicalis. *Methods Mol. Biol.* 1865, 1-17. 10.1007/978-1-4939-8784-9_1 30151755 PMC6421069

[DMM052169C40] Merscher, S., Funke, B., Epstein, J. A., Heyer, J., Puech, A., Lu, M. M., Xavier, R. J., Demay, M. B., Russell, R. G., Factor, S. et al. (2001). TBX1 is responsible for cardiovascular defects in velo-cardio-facial/DiGeorge syndrome. *Cell* 104, 619-629. 10.1016/S0092-8674(01)00247-1 11239417

[DMM052169C41] Moreno-Mateos, M. A., Vejnar, C. E., Beaudoin, J.-D., Fernandez, J. P., Mis, E. K., Khoka, M. K. and Giraldez, A. J. (2015). CRISPRscan: designing highly efficient sgRNAs for CRISPR-Cas9 targeting in vivo. *Nat. Methods* 12, 982-988. 10.1038/nmeth.3543 26322839 PMC4589495

[DMM052169C42] Park, B.-Y., Tachi-Duprat, M., Ihewulezi, C., Devotta, A. and Saint-Jeannet, J.-P. (2022). The core splicing factors EFTUD2, SNRPB and TXNL4A are essential for neural crest and craniofacial development. *J. Dev. Biol.* 10, 29. 10.3390/jdb10030029 35893124 PMC9326569

[DMM052169C43] Passos-Bueno, M. R., Ornelas, C. C. and Fanganiello, R. D. (2009). Syndromes of the first and second pharyngeal arches: a review. *Am. J. Med. Genet. A* 149A, 1853-1859. 10.1002/ajmg.a.32950 19610085

[DMM052169C44] Petit, F., Escande, F., Jourdain, A. S., Porchet, N., Amiel, J., Doray, B., Delrue, M. A., Flori, E., Kim, C. A., Marlin, S. et al. (2014). Nager syndrome: confirmation of SF3B4 haploinsufficiency as the major cause. *Clin. Genet.* 86, 246-251. 10.1111/cge.12259 24003905

[DMM052169C45] Ramirez, F., Ryan, D. P., Grüning, B., Bhardwaj, V., Kilpert, F., Richter, A. S., Heyne, S., Dündar, F. and Manke, T. (2016). deepTools2: a next generation web server for deep-sequencing data analysis. *Nucleic Acids Res.* 44, W160-W165. 10.1093/nar/gkw257 27079975 PMC4987876

[DMM052169C46] Rodríguez, J. I., Palacios, J. and Urioste, M. (1990). New acrofacial dysostosis syndrome in 3 sibs. *Am. J. Med. Genet.* 35, 484-489. 10.1002/ajmg.1320350408 2333875

[DMM052169C47] Rossi, A., Kontarakis, Z., Gerri, C., Nolte, H., Hölper, S., Krüger, M. and Stainier, D. Y. R. (2015). Genetic compensation induced by deleterious mutations but not gene knockdowns. *Nature* 524, 230-233. 10.1038/nature14580 26168398

[DMM052169C48] Sadaghiani, B. and Thiébaud, C. H. (1987). Neural crest development in the Xenopus laevis embryo, studied by interspecific transplantation and scanning electron microscopy. *Dev. Biol.* 124, 91-110. 10.1016/0012-1606(87)90463-5 3666314

[DMM052169C49] Saint-Jeannet, J.-P. (2017). Whole-mount in situ hybridization of Xenopus embryos. *Cold Spring Harb. Prot.* 2017, pdb.prot097287. 10.1101/pdb.prot097287 29084864

[DMM052169C50] Shaidani, N.-I., McNamara, S., Wlizla, M. and Horb, M. E. (2020). Animal maintenance systems: *Xenopus tropicalis*. *Cold Spring Harb. Protoc.* 2020, pdb.prot106146. 10.1101/pdb.prot106146 32404312 PMC7666031

[DMM052169C51] Shen, S., Park, J. W., Lu, Z.-X., Lin, L., Henry, M. D., Wu, Y. N., Zhou, Q. and Xing, Y. (2014). rMATS: robust and flexible detection of differential alternative splicing from replicate RNA-Seq data. *Proc. Natl. Acad. Sci. USA* 111, E5593-E5601. 10.1073/pnas.1419161111 25480548 PMC4280593

[DMM052169C52] Spokony, R. F., Aoki, Y., Saint-Germain, N., Magner-Fink, E. and Saint-Jeannet, J.-P. (2002). The transcription factor Sox9 is required for cranial neural crest development in Xenopus. *Development* 129, 421-432. 10.1242/dev.129.2.421 11807034

[DMM052169C53] Tanackovic, G., Ransijn, A., Thibault, P., Abou Elela, S., Klinck, R., Berson, E. L., Chabot, B. and Rivolta, C. (2011). PRPF mutations are associated with generalized defects in spliceosome formation and pre-mRNA splicing in patients with retinitis pigmentosa. *Hum. Mol. Genet.* 20, 2116-2130. 10.1093/hmg/ddr094 21378395 PMC3090192

[DMM052169C54] Trainor, P. A. and Andrews, B. T. (2013). Facial dysostoses: etiology, pathogenesis and management. *Am. J. Med. Genet. C. Semin. Med. Genet.* 163, 283-294. 10.1002/ajmg.c.31375 PMC3870197 24123981

[DMM052169C55] Trainor, P. A. and Krumlauf, R. (2000). Patterning the cranial neural crest: hindbrain segmentation and Hox gene plasticity. *Nat. Rev. Neurosci.* 1, 116-124. 10.1038/35039056 11252774

[DMM052169C56] Ulhaq, Z. S., Okamoto, K., Ogino, Y. and Ka Fai Tse, W. (2023). Dysregulation of spliceosomes complex induces retinitis pigmentosa-like characteristics in sf3b4-depleted zebrafish. *Am. J. Pathol.* 193, P1223-P1233. 10.1016/j.ajpath.2023.05.008 37263342

[DMM052169C57] Wieczorek, D., Newman, W. G., Wieland, T., Berulava, T., Kaffe, M., Falkenstein, D., Beetz, C., Graf, E., Schwarzmayr, T., Douzgou, D. et al. (2014). Compound heterozygosity of low-frequency promoter deletions and rare loss-of-function mutations in TXNL4A causes Burn-McKeown syndrome. *Am. J. Hum. Genet.* 95, 698-707. 10.1016/j.ajhg.2014.10.014 25434003 PMC4259969

[DMM052169C58] Will, C. L. and Luhrmann, R. (2011). Spliceosome structure and function. *Cold Spring Harb. Perspect. Biol.* 3, a003707. 10.1101/cshperspect.a003707 21441581 PMC3119917

[DMM052169C59] Wlizla, M., McNamara, S. and Horb, M. E. (2018). Generation and care of Xenopus laevis and Xenopus tropicalis embryos. *Methods Mol. Biol.* 1865, 19-32. 10.1007/978-1-4939-8784-9_2 30151756 PMC6396978

[DMM052169C60] Yamada, T., Takechi, M., Yokoyama, N., Hiraoka, Y., Ishikubo, H., Usami, T., Furutera, T., Taga, Y., Hirate, Y., Kanai-Azuma, M. et al. (2020). Heterozygous mutation of the splicing factor Sf3b4 affects development of the axial skeleton and forebrain in mouse. *Dev. Dyn.* 249, 622-635. 10.1002/dvdy.148 31900962

[DMM052169C61] Zahn, N., James-Zorn, C., Ponferrada, V. G., Adams, D. S., Grzymkowski, . J., Buchholz, D. R., Nascone-Yoder, N. M., Horb, M., Moody, S. A., Vize, P. D. et al. (2022). Normal Table of Xenopus development: a new graphical resource. *Development* 149, dev200356. 10.1242/dev.200356 35833709 PMC9445888

[DMM052169C62] Zhao, C., Bellur, D. L., Lu, S., Zhao, F., Grassi, M. A., Bowne, S. J., Sullivan, L. S., Daiger, S. P., Chen, L. J., Pang, C. P. et al. (2009). Autosomal-dominant retinitis pigmentosa caused by a mutation in SNRNP200, a gene required for unwinding of U4/U6 snRNAs. *Am. J. Hum. Genet.* 85, 617-627. 10.1016/j.ajhg.2009.09.020 19878916 PMC2775825

