# Supplementary material for: Deletion of sf3b4 causes splicing defects and gene dysregulation that disrupt craniofacial development and survival
Source: Dis Model Mech. 2025 Mar 24;18(3):dmm052169. doi: 10.1242/dmm.052169 (PMC11980789; doi:10.1242/dmm.052169)
Supplement: Supplementary information [file dmm-18-052169-s1.pdf]

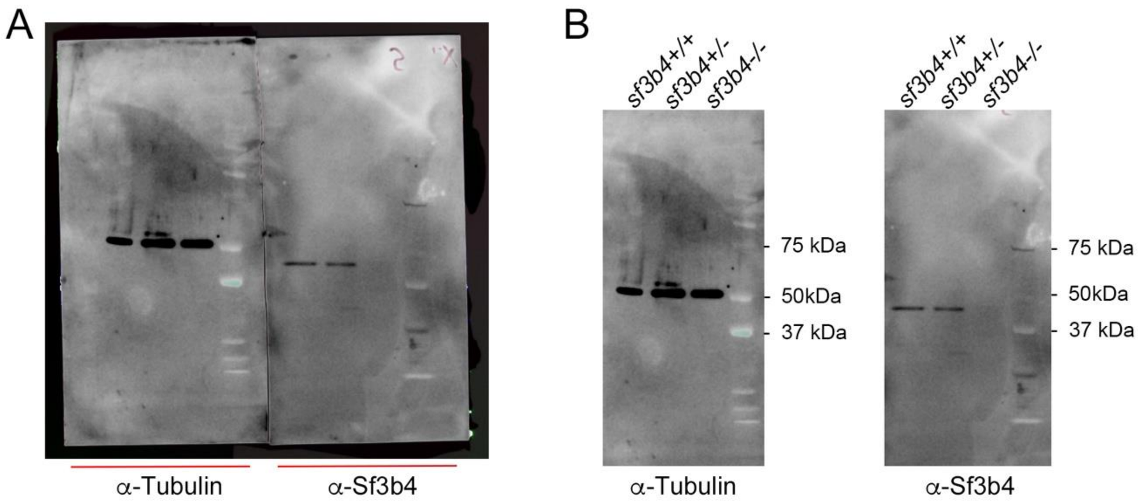

Fig. S1. Western blot analysis of protein extracts from wildtype and mutant embryos. (**A**) Original files of the raw unedited Western blots presented in Fig. 2C**.** (**B**) Annotated Western blot of the cropped versions presented in Fig. 2C**.**

Disease Models & Mechanisms • Supplementary information

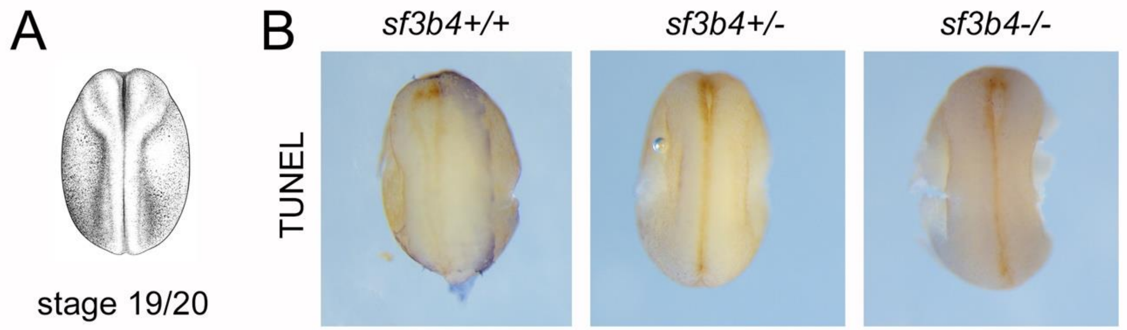

Fig. S2. TUNEL staining at neurula stage (NF Stage 19/20) comparing the 3 genotypes. (**A**) Neurula stage embryo at NF stage 19/20. Xenopus illustrations @ Natalyan Zhan (2022). (**B**) TUNEL staining of NF stage 20 embryos shows no difference across genotypes. In all panels, dorsal view, anterior to top.

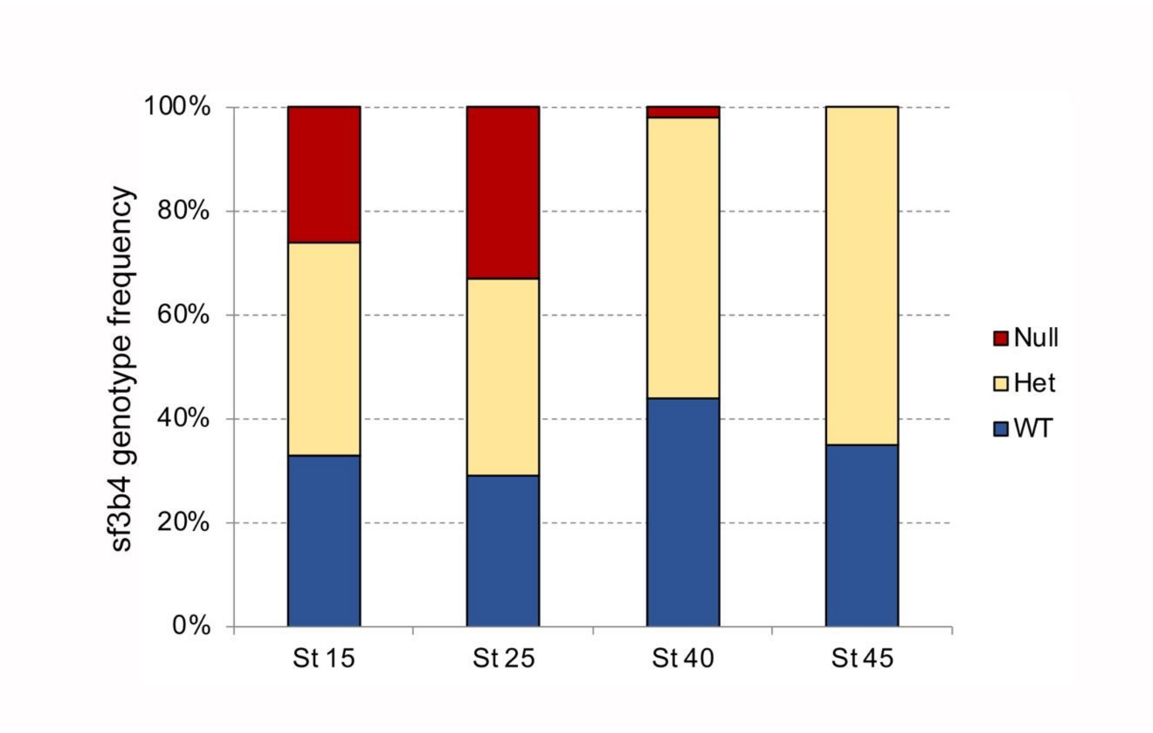

Fig. S3. Sf3b4 genotype frequency at different stages of development.

Disease Models & Mechanisms • Supplementary information

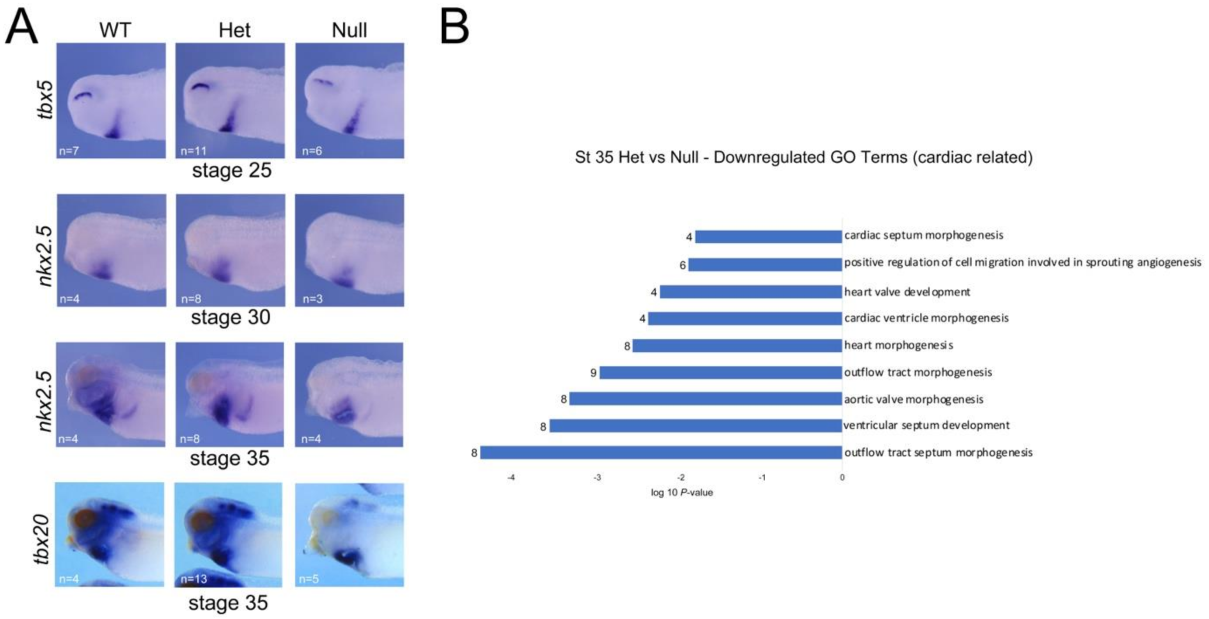

Fig. S4. Sf3b4 Null embryos exhibit cardiac defects. (**A**) Developmental expression of cardiac markers (*tbx5*, *tbx20* and nkx2.5) in WT and sf3b4 mutant embryos at stage 25, stage 30 and stage 35. Lateral views, anterior to left, dorsal to top. n, number of embryos analyzed per genotype. (**B**) GO term analysis for downregulated genes in Het vs Null at stage 35 shows an enrichment for cardiac terms.

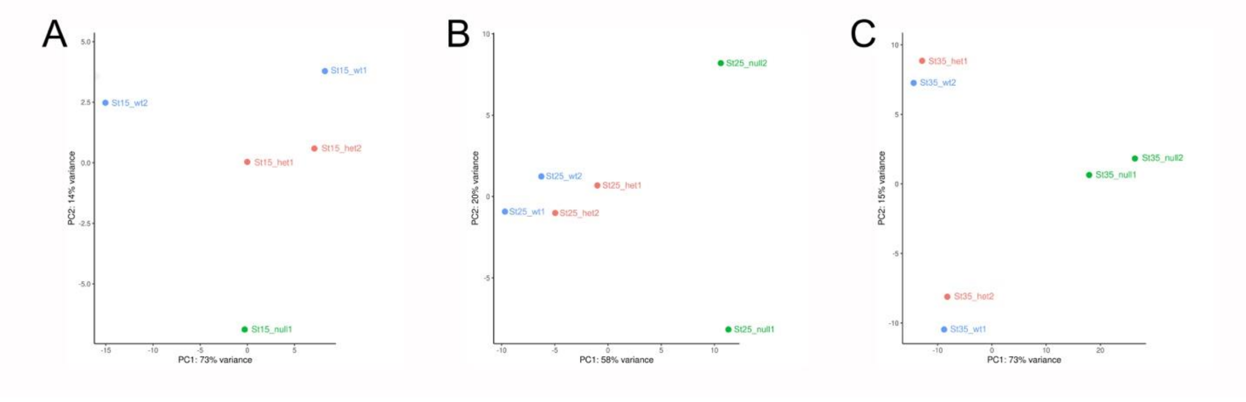

Fig. S5. Principal component analysis of WT and mutant *X. tropicalis* samples. At stage 15 (**A**), stage 25 (**B**) and stage 35 (**C**) WT and Het samples are more similar to each other than to the Null samples.

Disease Models & Mechanisms • Supplementary information

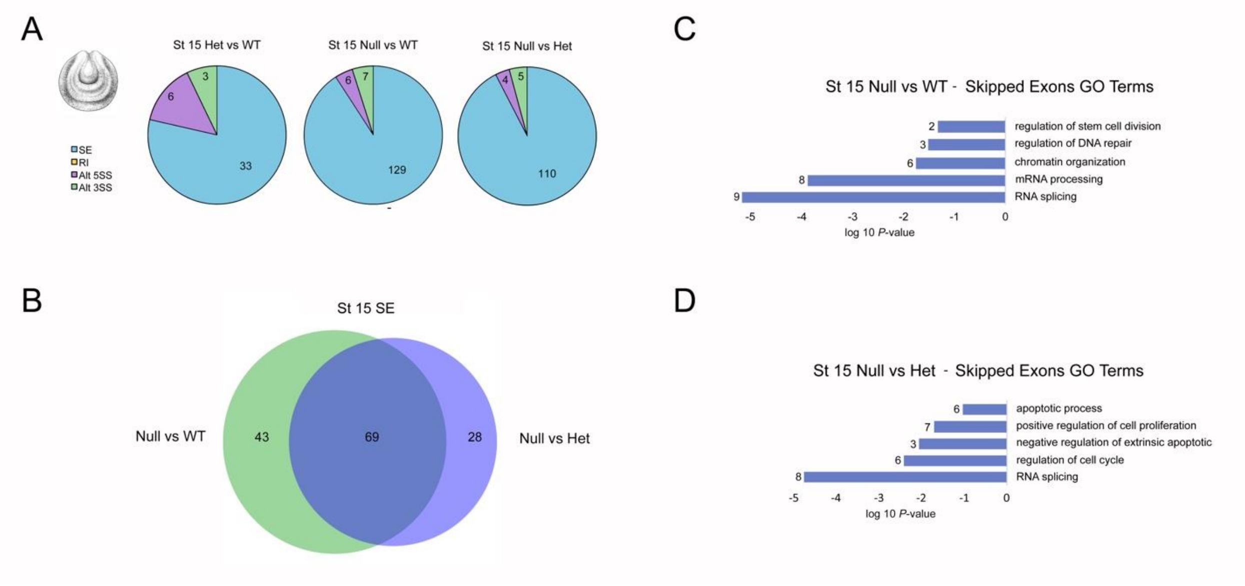

Fig. S6. RNA-seq analysis of splicing events in wildtype and *sf3b4* mutant embryos at stage 15. (**A**) Pie charts showing the type and number of splicing events occurring for each genotype comparison (Het vs. WT, Null vs. WT and Null vs. Het) at NF stage 15. Xenopus illustrations @ Natalyan Zhan (2022). (**B**) Venn diagram for genes with skipped exon at stage 15 (**C**). WT and Het samples show substantial overlap. (**C, D**) GO term analysis for genes with skipped exon at stage 15.

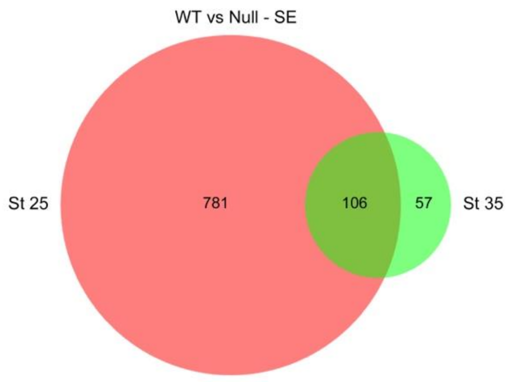

Fig. S7. Comparative analysis of genes with abnormal skipped exon events at stage 25 and stage 35. Venn diagrams illustrating that two thirds of the genes with aberrant skipped exon events at stage 35 also had skipped exon events at stage 25, that is 106 out of 163 in WT compared to Null (left panel), and 106 out of 159 in Het compared to Null (right panel).

Table S1. Developmental gene expression analysis per genotypes.

ISH Probe	Stage (NF)	WT	Het	Null
snai2	15	11	10	6
	15	5	5	3
sox10	15	5	9	5
	15	10	11	6
	15	15	13	9
tfap2e	15	3	11	7
sox2	15	5	8	6
sox10	20	3	8	4
sox9	25	8	6	5
sox9	25	6	7	5
sox10	25	2	6	6
	25	9	14	8
Sox9	40	10	16	0
	40	21	25	1
Runx2	40	9	8	1

Table S2. GO analysis of downregulated genes in *sf3b4* mutants at stage 35 shows an enrichment for cardiac terms.

Available for download at
https://journals.biologists.com/dmm/article-lookup/doi/10.1242/dmm.052169#supplementary-data

Disease Models & Mechanisms • Supplementary information

Table S3. Differentially expressed genes in WT vs Het at stage 25.

Available for download at
https://journals.biologists.com/dmm/article-lookup/doi/10.1242/dmm.052169#supplementary-data

Table S4. GO analysis of differentially expressed genes in *sf3b4* mutants at stage 25.

Available for download at
https://journals.biologists.com/dmm/article-lookup/doi/10.1242/dmm.052169#supplementary-data

Table S5. GO analysis of differentially expressed genes in *sf3b4* mutants at stage 35.

Available for download at
https://journals.biologists.com/dmm/article-lookup/doi/10.1242/dmm.052169#supplementary-data

Table S6. GO analysis of genes with aberrant skipped exons in *sf3b4* mutants at stage 25.

Available for download at
https://journals.biologists.com/dmm/article-lookup/doi/10.1242/dmm.052169#supplementary-data

Table S7. GO analysis of genes with aberrant skipped exons in *sf3b4* mutants at stage 35.

Available for download at
https://journals.biologists.com/dmm/article-lookup/doi/10.1242/dmm.052169#supplementary-data

Table S8. Comparison of genes differentially expressed in Null vs WT and Null vs Het for a sample of 20 genes at stage 25 and stage 35.

Available for download at
https://journals.biologists.com/dmm/article-lookup/doi/10.1242/dmm.052169#supplementary-data

Disease Models & Mechanisms • Supplementary information

Table S9. Primer sets used for qRT-PCR.

Gene	Forward primer	Reverse primer
phf5a	5'-GGCCGGCGAAGGAAAGGAAT-3'	5'-TACGCACAAGAGTGCACGGG-3'
eftud2	5'-ATCCCCACAGCCCGAAGAGT-3'	5'-GGGTGTGTGTTCGCAGGTCA-3'
sf3b3	5'-AGTGAGCCCTTGGAGGAGCA-3'	5'-TGTGCGTAGCGGAGCAAACA-3'
adamts1	5'-ACGCGAGTGCGATAACCCAG-3'	5'-CAACTGCAGGTCCACTGCCA-3'
mmp7	5'-CATGCCTATGGTCCAGGGCG-3'	5'-TGGCCAAATTCATGCGCAGC-3'
tbx1	5'-CGAGAGCGCAAAAGGCACAC-3'	5'-AAGGCAGGGACTGGCTGGTA-3'
ednrb	5'-AAACCGGTGGGCATAGGTGC-3'	5'-CCGTGTATGGGCAGCCACAA-3'
sema4c	5'-AGCAGGGAACCCGACTGACT-3'	5'-AGGAAAAGGGCCAGGCACAC-3'
eftud2 (exon 4)	5'-GGAGACCATAGTTCAAGAGGAAG-3'	5'-GTAACAGGTAATGTCTGCTCCAT-3'
ino80 (exon 30)	5'-ATCTTTGTGTTCCTGTTAAGCAC-3'	5'-CCAGTCGCTATCATAGAAAATCAC-3'
brd8 (exon 13)	5'-GCTGGATTTTGAGACTGTTGG-3'	5'-CACAAAATGACAGTGCTGCT-3'
ccna2 (exon 8)	5'-GACCTTCACCAGACCTACC-3'	5'-TGAGGAGAGAAACACCATGATAC-3'

Disease Models & Mechanisms • Supplementary information
